# Comparative transcriptome analysis of unripe and mid-ripe fruit of *Mangifera indica (var*. “Dashehari”) unravels ripening associated genes

**DOI:** 10.1038/srep32557

**Published:** 2016-09-02

**Authors:** Smriti Srivastava, Rajesh K. Singh, Garima Pathak, Ridhi Goel, Mehar Hasan Asif, Aniruddha P. Sane, Vidhu A. Sane

**Affiliations:** 1 CSIR- National Botanical Research Institute, Lucknow, 226001, India; 2 Academy of Scientific and Innovative Research (AcSIR), Anusandhan Bhawan, 2 Rafi Marg, New Delhi, 110001, India; 3 Umeå Plant Science Centre, Umea, Sweden

## Abstract

Ripening in mango is under a complex control of ethylene. In an effort to understand the complex spatio-temporal control of ripening we have made use of a popular N. Indian variety “Dashehari” This variety ripens from the stone inside towards the peel outside and forms jelly in the pulp in ripe fruits. Through a combination of 454 and Illumina sequencing, a transcriptomic analysis of gene expression from unripe and midripe stages have been performed in triplicates. Overall 74,312 unique transcripts with ≥1 FPKM were obtained. The transcripts related to 127 pathways were identified in “Dashehari” mango transcriptome by the KEGG analysis. These pathways ranged from detoxification, ethylene biosynthesis, carbon metabolism and aromatic amino acid degradation. The transcriptome study reveals differences not only in expression of softening associated genes but also those that govern ethylene biosynthesis and other nutritional characteristics. This study could help to develop ripening related markers for selective breeding to reduce the problems of excess jelly formation during softening in the “Dashehari” variety.

Mango (*Mangifera indica* L.) is one of the major fruit crops of the tropics and subtropics of Asia. It belongs to the family *Anacardiaceae* of order Sapindale and is known to evolve from tropical rainforests of south and south-east Asia^1^. The fruit is a favourite of masses due to its delicious taste, aroma and high nutritive value. Mango fruit is consumed in both raw and ripe forms^2^. India is the top Mango growing country and accounts for 42.06% of world’s production. Despite being the largest producer of fruit, India exported only 41,000 tonnes of Mango in 2013–14 as fresh fruit accounting for about 0.4% of production. In other words, its dominance as the largest producer is poorly translated into international trade. Due to faulty post-harvest practices during harvesting, packaging and storage approximately 20–25% of fruit are wasted. Major challenges affecting mango trade are short shelf life, high susceptibility to chilling injury, postharvest diseases and consumer demand for improved fruit quality. This wastage can be reduced to some an extent through proper understanding of fruit ripening using scientific methods.

Mango is an allotetraploid (2n = 40) with a predicted genome size of approximately 450 MB^3^. The mango genome sequence is not available yet. However, with the advent of next generation sequencing techniques like Illumina HiSeq2000 and Roche 454, some reports have started pouring in to enrich our knowledge about the mango transcriptome. Wu *et al.*, in 2014 have used Chinese variety “Zill” and performed transcriptomic and proteomic analysis utilizing 4 different developmental stages of fruit^4^. Although, this study deals with fruit ripening it does not provide any concrete information regarding gene families involved in fruit ripening. Dautt-Castro *et al.* have also reported a transcriptomic study using mango fruit mesocarp from “Kent” variety^5^. They found a differential expression pattern of several ripening related genes between unripe and ripe mangoes. Azim *et al.*, have sequenced the mango chloroplast genome^6^. The group used “*Langra”* variety of mango and obtained a circular map of the chloroplast genome with 151,173 basepairs but they failed to obtain a complete chloroplast DNA sequence.

India is a land of mangoes with nearly 1,000 different varieties grown in different parts of the country; however, 20 varieties are grown commercially. Commercial varieties that are grown in India include Alphonso, Dashehari, Langra, Chausa, Banganapalli, Kesar etc. “Alphonso” is the main variety which is exported. “Dashehari” is very popular in northern India as it is fibreless, delicious in taste with mild aroma and has a very high pulp to stone ratio. However, the consumption of this variety is limited majorly to India because of the rapid and uneven ripening. “Dashehari” ripens from the stone towards the periphery. There is jelly formation in the pulp near the stone at late ripe stage although it looks firm and good from outside. Our group has been working on “Dashehari” ripening for the last few years to understand ripening process^7^,^8^,^9^,^10^,^11^. In the present study we report a detailed comparative transcriptomic analysis between two different stages (unripe and mid ripe) of ripening. Since jelly is formed in inner parts of the fruit we have chosen inner zones of unripe and ripe “Dashehari” fruit for transcriptomic analysis. For the real time validation of our data we have taken three stages unripe, midripe and ripe mangoes of “Dashehari”. From the present study several differentially expressed genes were identified that govern the process of ripening in “Dashehari” mangoes.

## Results and Discussion

**Figure 1:**
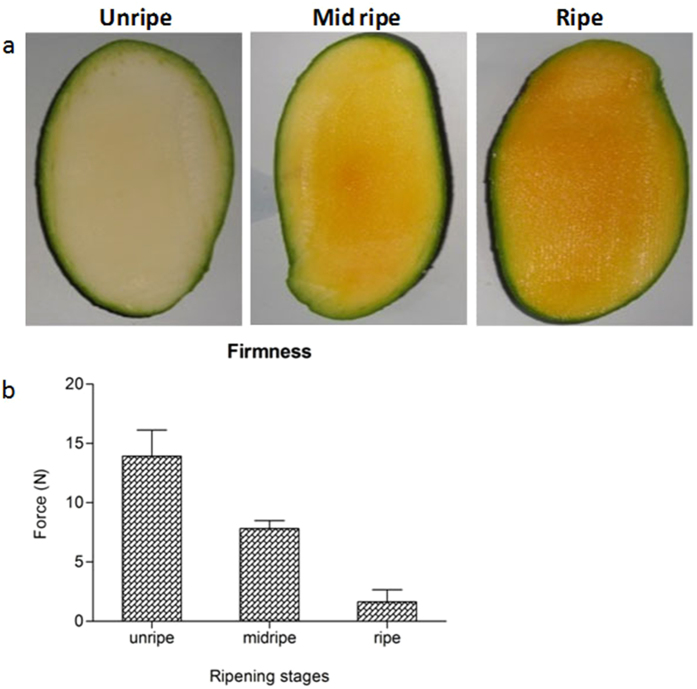
(**a**) Pictorial representation of ripening stages of “Dashehari” mango fruit where progression of ripening from stone to peel is shown. (**b**) Firmness of mango fruit (*var*. “Dashehari”) during different stages of fruit ripening, measured using penetrometer. All the measurements were done in triplicates and data is represented as mean + SE.

Ripening process of “Dashehari” mango was monitored during post harvest at room temperature. The colour of the fruit pulp changed gradually from whitish to dark yellow with the progression of ripening (Fig. 1a); however, peel colour remained green. The colour change was also accompanied with a decrease in fruit firmness from 13.9 ± 2.19 N in unripe fruit to 1.6 ± 1.01 N in the ripened fruit (Fig. 1b).

### Transcriptome sequencing and assembly

**Figure 2:**
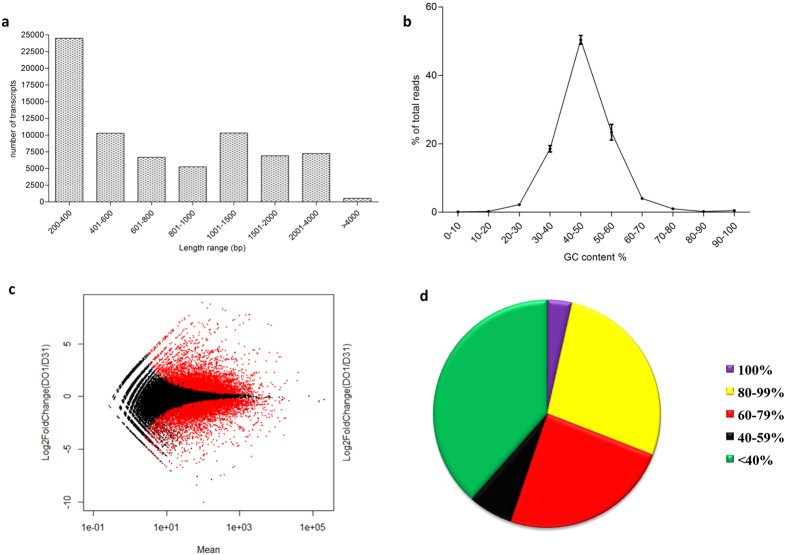
General features of “Dashehari” transcriptome. (**a**) Length distribution of *M. indica* transcripts from the combined assembly. (**b**) GC content distribution. (**c**) Fold change and mean plot between unripe inner zone and midripe inner zone samples using DESeq program. The transcripts with p-value < 0.01 are shown in red. (**d**) Blastx similarity score distribution of “Dashehari” transcriptome with NR database.

The transcriptome of the fruit pulp of inner zones of unripe and mid ripe stages of “Dashehari” mango was sequenced using Illumina Hi-Seq and 454 Titanium GS-FLX. The sequencing was carried out in triplicates. The raw reads were quality filtered and assembled using the Trinity programme. 24,339,498 raw reads obtained from Illumina sequencing were trimmed down to 23,154,701 reads using Trimmomatic tool. The alignment summary of reads on assembled transcriptome is given in Table 1. Overall 74,312 unique transcripts with ≥1 FPKM were obtained. Hybrid assembly was carried out with the trimmed reads (from 454 pyrosequencing and from Illumnina Hi-Seq) using Trinity. A total of 71,733 contigs were assembled with an average length of 942 bp (Fig. 2a) and 44.6% GC content (Fig. 2b). The transcripts with p-value <0.01 (from DESeq program) are shown in red (Fig. 2 c). On the whole 46,412 (~74.68%) of assembled transcripts had at least one significant hit in NCBI NR database. Around 59% of the transcripts found using Blastx had confidence level of at least 1e^−10^, which indicates high protein sequence similarity (Fig. 2d).

### Annotation and functional characterisation

**Figure 3:**
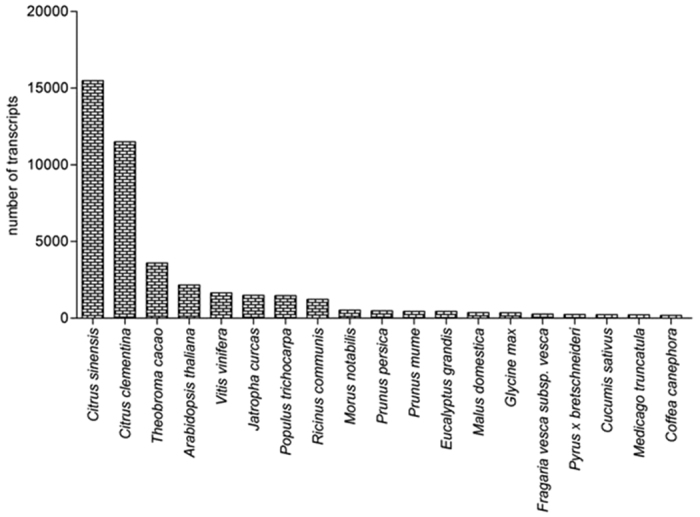
Blastx top 15 hit with different organisms: Mango transcriptome exhibits maximum similarity with *Citrus sinensis* along with *Citrus clementina*.

**Figure 4:**
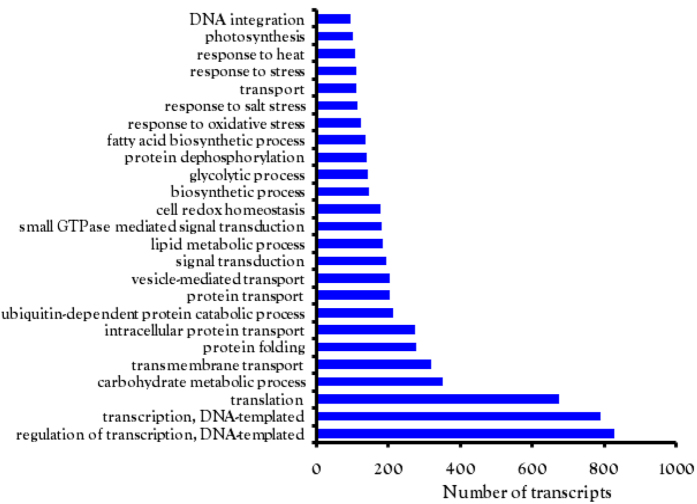
Top 25 terms in biological process category from GO annotation: the bars on x axis represent the number of transcripts for the biological processes mentioned on the y axis.

The total transcripts were annotated against UniProt and NCBI NR database. According to the UniProt and NR databases 34,934 and 44,472 genes were annotated respectively. A total of 25,614 contigs showed no significant hits in any of the database and could be novel mango specific genes. The transcriptomes from other varieties of mango have been reported from pulp and pericarp of “Zill” mango, mesocarp of “Kent” variety and leaf of “Langra”^4^,^5^,^6^. A similar number of genes were also reported in other transcriptomic studies carried out in pulp tissue of “Zill” and “Kent” varieties (Table 2). Smaller number of unigenes was reported in leaf transcriptome of “Langra” variety of mango^6^. This suggests that a large number of genes are conserved and active in different varieties of mango. Going further with our results, blastx against NR database was performed at *E*-value 10^−10^. Most of the top hits were from *Citrus* sp with *Citrus sinensis* and *Citrus clementina* being the top two hits out of 15 organisms (Fig. 3). A total of 59.05% genes were annotated by Citrus sp. (approximately 26,000 out of total 44,472 contigs showed matches with Citrus sp). The order of the top 15 organisms varied from the data of “Kent” variety of Mango indicating differences in the sequences of different varieties^5^. In this study we compared our “Dashehari” transcriptome data mainly with “Kent” data because both varieties have different natural habitat, “Kent” being an American one whereas “Dashehari” is a North Indian variety. The time period of ripening of both varieties is different, “Dashehari” ripens in summers and “Kent” is available during monsoons. The ripened “Dashehari” is green from the outside (peel is green) but in the case of “Kent” variety, the peel colour of ripened mango varies from being greenish red or yellow. We compared the assembled data of “Kent” variety with the total transcripts obtained from our study and found 58,934 contigs matched with “Kent” variety with sequence identity of 97% and 12,799 contigs were specific to the “Dashehari”. The “Dashehari” transcripts were also annotated using GO terms and 22,632 transcripts mapped to 45,262 significant GO terms. These were divided into the three categories i.e., biological processes, molecular function and cellular components. The maximum number of GO terms mapped to regulation of transcription followed by translation and carbohydrate metabolism (Fig. 4). According to the eggNOG database 1,109 genes showed functional annotation. The highly expressed protein families belonged to protein kinases, cytochrome P450s, mitochondrial carrier proteins, protein phosphatases among others, indicating active metabolism in mango fruit during ripening. A total of 47 plant transcription factor families were identified when queried against the plant transcription factor database. Of these the MYB, bHLH, WRKY, HSF, NAC, bZIP, ERF were some of the highly expressed transcription factors. Differential regulation of gene family members was observed in nearly all of the plant transcription factor families (Supplementary Table S1).

The KEGG analysis showed that the genes for most of the pathways could be mapped to the mango transcriptome and the transcripts related to 127 pathways were identified in the “Dashehari” mango transcriptome. The maximum number of matches was from the *Citrus clementina* pathway and genes for many of the pathways were mapped for over more than 90% coverage. The pathway enrichment analysis was done using the KOBAS v2.0 and 37 pathways were significantly enriched with a p-value of <0.1. These pathways ranged from detoxification, ethylene biosynthesis, carbon metabolism and aromatic amino acid degradation (Supplementary Table S2).

### Differential gene expression and functional characterisation

Fruit ripening is a complex process and requires the coordinated expression of a wide number of genes. At this stage the fruit is metabolically active and a large number of genes are differentially expressed. The ripening process involves drastic changes in expression of genes related to ethylene biosynthesis and signal transduction, cell wall loosening and expansion, flavour and pigment formation. The differential gene expression was studied in the unripe and midripe stages of “Dashehari” fruit. 1447 genes were up-regulated (>2 fold, p-value <0.05) and 2003 genes were down-regulated (≤2 fold, p-value <0.05) in midripe stage as compared to unripe stage. The top 100 differentially expressed genes are represented in Supplementary Fig. S1. The GO analysis of the differentially regulated genes revealed that genes for the metal ion binding, cell wall metabolism, oxidoreductase activity among others were up-regulated and genes for abiotic stress, transport, carbohydrate metabolism were down-regulated (Table 3).

### Genes related to ethylene biosynthesis and signaling

**Figure 5:**
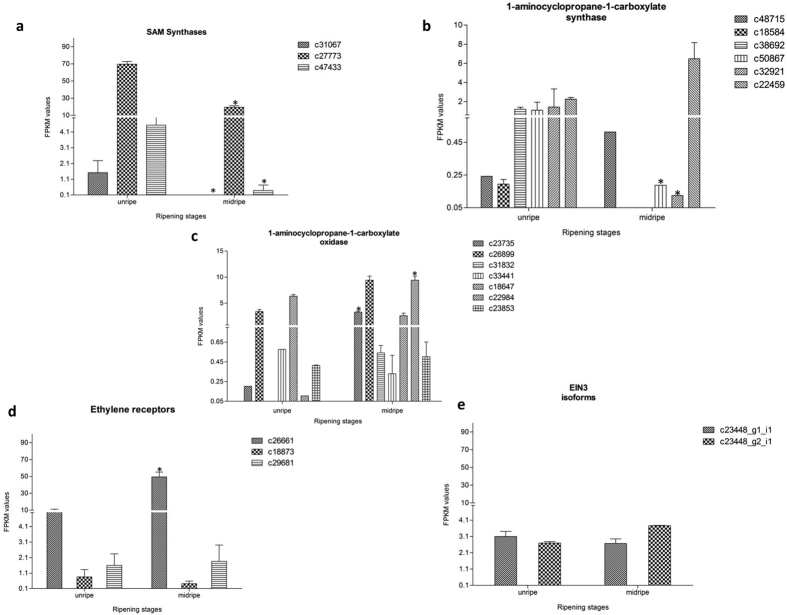
Digital expression profiles of contigs belonging to ethylene signaling pathway from two different stages of “Dashehari” fruit ripening. (**a**) SAM synthatases, (**b**) 1, aminocyclopropane-1-carboxylate synthases (ACS), (**c**) 1-aminocyclopropane-1-carboxylate oxidases (ACO), (**d**) Ethylene receptors, (**e**) EIN3 isoforms. Illumina sequencing was performed in triplicates, data is represented as mean with +SE. P = 0.01 for all data sets except for EIN3 for which P = 0.5. *On bars shows significant difference at P = 0.01.

S-adenosyl methionine (SAM) is the most important methyl contributor in plant system and many biochemical pathways^12^. The first committed step of ethylene biosynthesis is the conversion of SAM to 1-aminocyclopropane-1-carboxylic-acid (ACC) by ACC synthase. Thus SAM acts as the precursor for ethylene biosynthesis^13^,^14^. Nearly 80% of the total cellular methionine gets converted to SAM by the action of SAM synthetase at the expense of ATP^12^. Three SAM synthetases (c31067, c27773 and c47433) were identified from “Dashehari” transcriptome. All of them were 2–3 fold down-regulated in mid ripe stage as compared to unripe one (Fig. 5a).

ACC synthase (ACS) and ACC oxidase (ACO) are the key regulators of ethylene biosynthesis in plants and changes in the expression of these genes govern the ethylene production. In this study 6 genes for ACS were identified (Fig. 5b). Only one of these six (c18584_g2_i1) was full length and the rest (five) were partial homologs, out of which c50867 and c32921 showed 2–3 fold down-regulation (Supplementary Fig. S2a). In the “Kent” variety, two ACSs were identified with no change in expression profile between ripe and unripe stage^5^. No significantly up-regulated ACS homolog was found in the transcriptome study. Similar to our findings, none of the ACS homolog was found differentially up-regulated in ripe mango in “Kent” transcriptome. It is possible that ACS homologs are differentially up-regulated in early ripening stages when ethylene is required to start ripening process. In our case we have used mid ripe stage (3^rd^, 4^th^ day post harvest) and it is possible that ACS peak was attained on day one. Citrus, showing maximum similarity with mango transcriptome, also has 6 ACSs (information gathered from Citrus Genome Database). Nine ACS genes (*LeACS1A*, *B* and *LeACS2* to *LeACS8*) have been reported from tomato^15^, where only *LeACS1A*, *LeACS2*, *LeACS4*, and *LeACS6* were expressed in mature and ripening fruit with differential transcript patterns^16^,^17^. By exploring the apple whole genome in 2013, Li *et al.* identified 19 *ACS* genes^18^. Out of these, six genes were expressed only in fruit. They speculated that different ACSs specifically expressing in fruit might further be categorised to work in System 1 or system 2 of ethylene biosynthesis^18^.

Seven contigs related to ACO were identified in the “Dashehari” transcriptome data. ACO is a terminal enzyme of ethylene biosynthetic pathway. Out of these 7contigs only c23735 and c22984 were significantly up-regulated (Fig. 5c, Fig. S2b). Three ACC oxidase genes were reported to up-regulate in ripe “Kent” mango^5^. Mango ACO genes, therefore, also belong to a small gene family like Arabidopsis, Citrus (5 ACOs each) and tomato (6 ACOs)^19^,^20^. ACOs from different fruits have been shown to have tissue and developmental specific expression. One of the three identified isoforms of ACO gene *Md-ACO1* in apple (*Malus domestica* cv. Royal Gala), has been shown to be restricted to fruit tissues, with optimal expression in ripening fruit^21^. The studies on climacteric fruits (tomato and banana) have shown a differential feedback regulation of ACS and ACO between pre-climacteric and ripening fruits and also between different tissues^16^,^22^,^23^.

The ethylene signal is perceived and transmitted by complex signal transduction pathway involving many genes. These include the ethylene receptors and ethylene responsive transcription factors that ultimately result in regulation of ethylene related genes. In Arabidopsis there are 5 ethylene receptors and one CTR^19^, in tomato 8 ethylene receptors and 3 CTR^20^ and in Citrus 9 ethylene receptors have been reported (from citrus genome database). We could dig out 3 ethylene receptors (c26661, c29681 and c18873) from “Dashehari” transcriptome. Out of these 3 genes, contig c26661 was significantly up-regulated (~2 fold) in the mid ripe stage of fruit ripening (Fig. 5d). The two ERSs (c29681 and c18873) were also identified but change in expression was not significant in mid ripe stage as compared to unripe stage. Three ethylene receptors (two ETRs and one ERS) were also reported in the “Kent” variety of which two ETR homologs remained unchanged whereas one ERS showed down-regulation^5^. In a recent report, different versions of the mango ethylene receptor *MiERS1* from Hôi’ cultivar were identified by Winterhagen *et al.*^24^. They suggested that in addition to *MiERS1*, two short versions of this receptor (*MiERS1m*, *MiERS1s*) are also present in mango. These short versions reveal deletions of crucial domains for protein function. Authors speculated that the short versions of *ERS*s might play a role either in the CTR1-dependent pathway by modifying the signal output in receptor complexes or via alternative signalling pathways.

Two contigs related to *CTR1* were identified in the transcriptome data though none of these showed any significant change in the expression during progression of ripening (Supplementary Fig. S3). In the variety “Kent”, only one *CTR* has been reported which shows down-regulation in ripe fruit with respect to unripe fruit. Arabidopsis has one *CTR1*. Interestingly, CTR1-like proteins (*LeCTRs*) in tomato are encoded by multigene families^20^. All these *LeCTRs* could interact with ethylene receptors. Detailed studies are required to find out role of multiple *CTRs* and whether the different *LeCTRs* are functionally redundant or have unique roles in ethylene signalling^20^.

We also found two isoforms of *EIN3*- a positive regulator of ethylene signalling pathway. Contig 23448_g2_i1 encoding EIN3 was up-regulated while the other was down regulated (23448_g1_i1) at P = 0.5 (Fig. 5e). The presence and changes in the expression patterns of genes of ethylene signalling pathway between “Dashehari” and “Kent” indicate varietal differences and changes in ripening process between these two varieties. There were 44 ethylene responsive transcription factor genes that we observed in the total transcriptome, of these 39 belonged to the ERF family and 5 to the RAP family.

Plant 14-3-3s are conserved proteins that bind to a range of transcription factors and play role in many hormonal signal transduction pathways. In “Dashehari” transcriptome 10 contigs related to 14-3-3 were identified, however, only two genes showed significant differential expression in mid ripe stage (Supplementary Fig. S3). These proteins in recent years have been shown to affect ethylene signaling pathways^25^. 14-3-3 proteins are shown to interact with (CTR1) in Arabidopsis. Rice and Arabidopsis 14-3-3 protein are associated with ACS^26^,^27^. Besides rice and Arabidopsis, four 14-3-3 proteins have been identified from banana and based on transcript analyses *Ma-14-3-3a* and *Ma-14-3-3e* genes were shown to be involved in regulating ethylene biosynthesis during ripening^28^.

### Genes related to fruit softening

One of the most important changes occurring during ripening is loss of firmness. This softening is due to the loosening of the cell wall and degradation of the lamella mainly composed of pectins^29^. The important cell wall loosening and degrading enzymes/proteins are Expansin (EXP), Polygalacturonase (PG), Pectate lyase (PL), Pectin methyl esterase (PME), Xyloglucan endoglucosyltassferase (XTH), endoglucanases and galactosidases etc. A total of 60 cell wall related genes were identified in the transcriptome of “Dashehari” mango and were differentially expressed. A large number of EXPs, XTHs and PLs showed differential transcript levels in unripe and midripe stages of mango.

**Figure 6:**
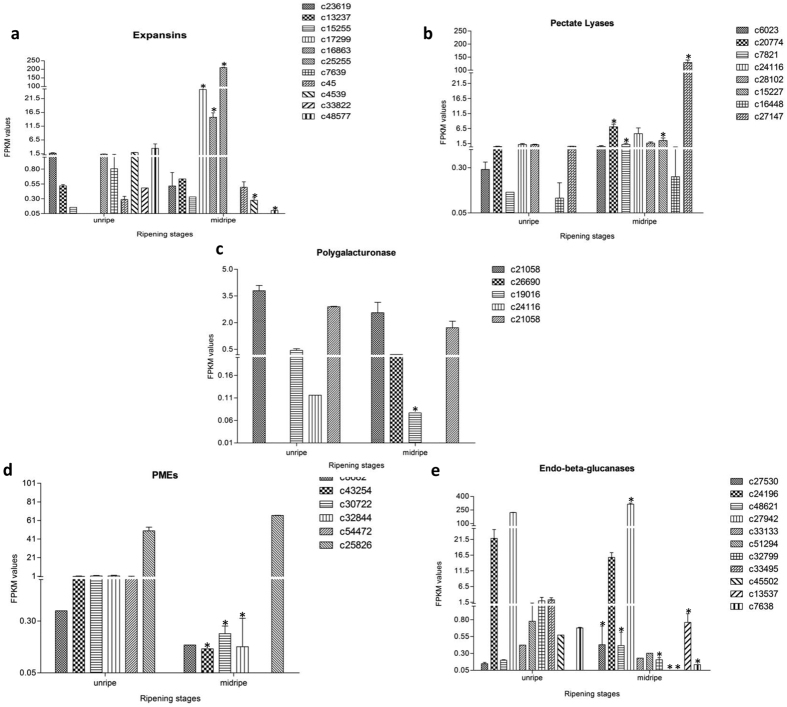
Digital expression profiles of contigs belonging to Cell wall modifying proteins from two different stages of “Dashehari” fruit ripening. (**a**) Expansins, (**b**) Pectate Lyases (PL), (**c**) Polygalacturonases (PG), (**d**). Pectinmethyl esterases (PME), (**e**) Endo-beta-glucanases. Illumina sequencing was performed in triplicates, data is represented as mean with +SE. P = 0.01 for all data sets. *On bars shows significant difference at P = 0.01.

Eleven expansin sequences were detected in the “Dashehari” transcriptome of which 3 expansin genes (contigs c25255, c17299, c16863) showed differential >2 fold up-regulation (7, 5, 5 fold respectively). Contig c25255 matched 100% with previously characterised ripening related expansin (*MiExp1*) from mango^7^. c48577, c4539 encoding expansins on the other hand were down-regulated (5 and 2 fold respectively). The rest of the expansin genes did not show much change in expression during unripe and midripe stages of mango. Dautt-Castro *et al.* also reported five expansins which were up-regulated in ripe “Kent” fruit^5^. In Zill mango, also expansin genes had increased expression during fruit ripening^4^. It is possible that besides *MiEXP1* a minimum of two more expansins play a role in ripening associated softening. The down-regulated expansin genes might be involved in fruit development rather than ripening (Fig. 6a). Five expansins are reported in banana and only 2 (*MiEXP1 and 2*) play major role during ripening^30^. Multiple genes related to fruit softening have also been identified from other dicot fruits such as apple, pear and strawberry. Simultaneous expression of multiple genes of the same family during softening suggests complexity of the softening process.

Pectins are major component of mango cell wall. Chaurasia *et al.* suggested that the pectate lyases could be the most important pectin degrading enzymes in mango and *MiPel1* expression levels increased during the course of fruit ripening^8^. Transcriptome sequencing of mango suggested that there are at least 8 PLs and pectate lyase like genes of which 4 contigs (c7821, c20774, c15227, c27147) were up-regulated during ripening and the rest did not show any significant changes (Fig. 6b). Dautt-Castro *et al.*^5^ also reported 4 pectate lyases in the “Kent” variety^5^. In the current study contig c27147 corresponding to *MiPel1* also showed increased expression in mid ripe stage as compared to unripe stage. The other pectin degrading enzyme polygalacturonse (PG) was also studied. In this study 5 PG genes (Fig. 6c) were identified. Except for c19016, expression of other homologs remained unchanged in unripe and mid ripe stages of “Dashehari”. Interestingly, c19016 was significantly down-regulated at mid ripe stage. In an earlier study carried out in the lab “*Dasheahri”* had very little PG enzyme activity^31^. This is in contrast to the observation made by Dautt-Castro *et al.*, for the “Kent” variety^5^. The group reported 9 PG genes with significant up-regulation of six unigenes encoding PG during ripening. The changes in PG transcripts could possibly be due to the subtle changes in cell wall composition of the two varieties. We speculate that in “Dashehari”, PL might be the major pectin solubilising enzyme instead of PG and could be a reason of jelly formation at late ripe stage. Six genes encoding for PMEs were present in “Dashehari” transcriptome of which 3 (c32844, c30722, c43254) were 2–3 fold down-regulated (Fig. 6d). The activity of pectin methylesterase precedes the activity of PL and PG, hence it might be required during the early phase of transition to ripening and once the PL and PG become active the PME expression goes down. Endo-beta-glucanases are also considered important enzymes related to cell wall loosening. In the transcriptome of “Dashehari” fruit, 11 endo-beta-glucanases were identified, of these five were up-regulated during ripening (c27530, c48621, c27942, c45502 and c13537). Three genes were down-regulated (c32799, c33495, c7638) indicating their possible role more in fruit development rather than ripening (Fig. 6e). Contig id c27942 has identical sequence to that reported for previously characterised *MiCel1* from “Dashehari” and also shows the same expression profile^9^.

Xyloglucan endotransglycosylase and transferases are important cell wall modifying enzymes which play important role in softening process of fruits like banana, tomato, apple etc. In the present study 17 genes were identified, of these two belonged to Xyloglucan transferase category and the rest were endotransglycosylases. Both xyloglucan transferases were down-regulated in the ripe stage. Out of 15 endotransglycosylases, three genes were up-regulated (c6594, c23621, c22673) and 3 were down-regulated (c4555, c49565, c38502), the rest did not show much change in their expression during ripening (Supplementary Fig. S5). The expression of XTHs was, however, not significantly changed in “Kent” variety^5^.

A total of 26 galactosidase genes were identified in “Dashehari” fruit, of these 5 were alpha and the rest were beta galactosidases. Two beta-galactosidase genes were up-regulated (c28255, c23121), two were down-regulated (c30645, c21739) and the rest did not show any change in expression. Two alpha-glucosidase genes were significantly down-regulated (c9499, c21528), the expression of rest of the homologs remained unchanged (Supplementary Fig. S6). Unlike the “Kent” variety, galactosidase genes in “Dashehari” did not show significant differential change. No rhamnogalactouronan gene was identified in the “Dashehari”; however, in the “Kent” variety the RGL1 gene was highly expressed during ripening^5^. The differential expression changes in various cell wall modifying enzymes in “Dashehari” and “Kent” varieties further suggest differences in cell wall composition of these two varieties and need to be studied in detail in order to develop varieties with better shelf life.

### Genes related to flavour and colour

**Figure 7:**
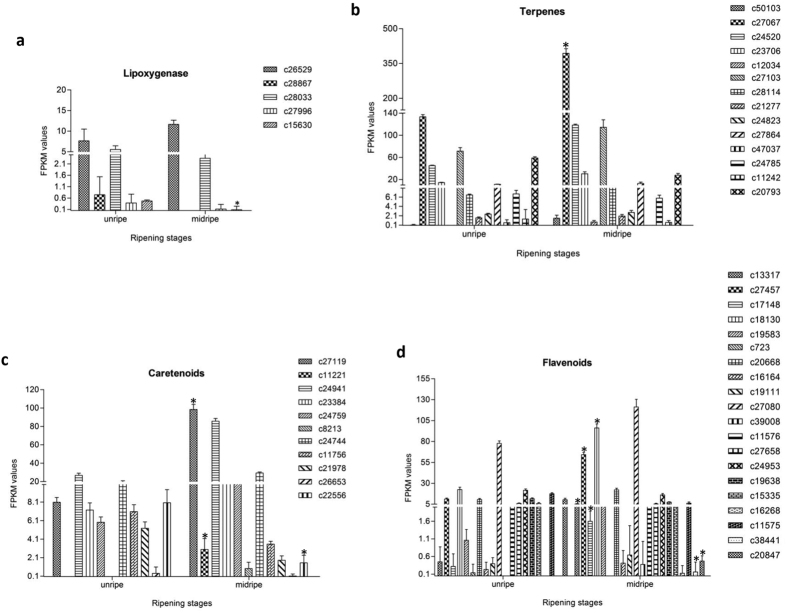
Digital expression profiles of contigs belonging to different aromatic pathways from two different stages of “Dashehari” fruit ripening. (**a**) Lipoxygenases (**b**) Terpenes (**c**) Carotenoids, (**d**) Flavonoids. Illumina sequencing was performed in triplicates, data is represented as mean with +SE. P = 0.01 for all data sets. *On bars shows significant difference at P = 0.01.

Flavour (aroma and taste) is due to the blending of acidity and aroma volatiles, sweetness, saltiness and acidity present in the fruit. There are 578 volatile compounds reported from mango fruit in various cultivars^32^. The presence of large number of varieties of mango that differ in aroma makes mango an interesting system for the study of aroma. About 54 contigs related to flavour showed significant differential regulation during ripening of “Dashehari” mango. The LOX pathway genes are also involved in the aroma development. Five contigs belonging to lipoxygenase pathway were indentified in the “Dashehari” transcriptome (Figs 4 and 7a (c28867, c28033, c27996 and c15630) were down-regulated in mid ripe stage while transcript levels of c26529 increased in mid ripe stage. On the contrary 4 Lox genes in the “Kent” variety^5^ were significantly up-regulated in ripe fruit. Mango fruit vastly differ in aroma volatiles. The changes in the transcript profiles of genes of LOX pathway between Kent and “Dashehari” again indicates the involvement of different aroma pathways in different varieties. 14 contigs related to terpenoid pathway were also identified (Fig. 7b). The farnesyl pyrophosphate synthase (FPPS) and geranylgeranyl pyrophosphate synthase (GGPS) were shown to be involved in the aroma volatiles of “Kent” variety; however, in the present study the FPPS and GGPS genes were not significantly up-regulated indicating lesser role of these genes in the flavour development of “Dashehari” mango. From the carotenoid pathway out of 11 contigs, 7 contigs were significantly up-regulated and 4 were down-regulated in mid ripe stage (Fig. 7c). Contig related to phytoene synthase (PSY, c27119) was up-regulated in midripe fruit of “Dashehari”. In “Kent” contig related to PSY was highly expressed but did not change during ripening. Three contigs related to 9-cis-epoxycarotenoid dioxygenase were also present in “Dashehari” transcriptome. Contigs c53134 and c1330 were 2 to 3 fold down regulated whereas c27658 was four fold up-regulated (Supplementary Table S3). Chalcone synthase was not identified in “Dashehari”, however, it was present in “Zill” mango. We found 20 contigs fitting into the flavonoid pathway out of which 10 were upregulated and 10 were down-regulated (Fig. 7d). Amongst the up-regulated ones c13317, c27457, c17148 and c18130 showed significant changes while c38441 and c20847 were significantly down-regulated in midripe stage.

Mango is an excellent source of β-carotene and α-tocopherols (www.ars.usda.gov/nutrientdata). The precursor substrate for both these phytonutrients is p-hydroxyphenylpyruvate (p-HPP). HPP is converted to homogentisate (HGA) by the enzyme p-hydroxyphenylpyruvate dioxygenase (HPPD). HGA is then utilized through two different pathways to form tocopherols and carotenes^33^,^34^. In a previous study, Singh *et al.* reported characterization of HPPD gene from mango. *MiHPPD* expression was ripening-related and correlated with an increase in tocopherol and carotenoid content in mango fruit^11^. The expression was triggered with the initiation of ripening. Therefore, contigs showing sequence similarity to *MiHPPD* were searched in the transcriptome data. Two contigs (c27898_g1_i2 and c27898_g1_i3) showed similarity with *MiHPPD* (Suppplementary Table S4). Contigs c27898_g1_i3 and c27898_g1_i2 had 95–98% identity with *MiHPPD* sequence. In transcriptome data these did not show significant change between unripe and mid-ripe stages. It is possible that the transcript level of *MiHPPD* level increased post mid-ripe stage and did not show much change during early ripening.

Mono- and/or sesquiterpene hydrocarbons are the predominant constituents of Indian mango cultivars. However, levels of other volatiles like alcohols, aldehydes, esters, furanones and lactones also play role in imparting specific aroma are also responsible for the distinct flavour and taste of different mango cultivars^35^. Alcohol dehydrogenase (ADH) genes are expressed in a developmentally regulated manner particularly during fruit ripening and have been shown to play a major role in flavour development in ripe tomato, melon^36^. In Dashehari transcriptome data 8 contigs related to ADH were identified. Three showed similarities to the already identified *MiADH*s from mango^10^. These *MiADH*s shared high homology at nucleotide and amino acid level in the coding region. These contigs were down-regulated in midripe stage (<2 fold change, not significant). Singh *et al.* also reported that all three *MiADH*s were highly up-regulated on day one post harvest and then the transcript abundance decreased further during progression of ripening^10^. The promoter regions of these genes were divergent suggesting that the three ADH genes might be under regulation of different cues though present in ripening fruit^10^. Besides these, additional four ADHs present in the transcriptome did not show significant changes in their expression level.

### Real time validation of differentially expressed genes

**Figure 8:**
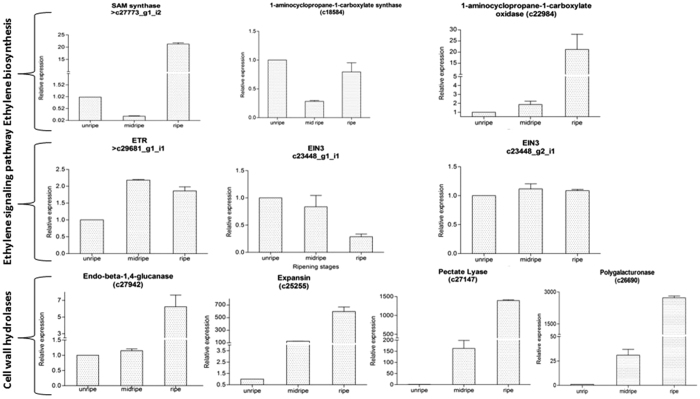
Real time validation of genes selected from various pathways: Quantitative Real time PCR of representative contigs from different gene families was carried out using total RNA isolated from inner zones of unripe, mid ripe and ripe fruit pulp tissue of “Dashehari” mango. Experiment was performed in triplicates and data is represented as +SE.

In order to validate transcriptomic data (although sequencing was carried out with three biological replicates) some representative genes from various pathways were selected for real time PCR validation. The expression pattern during the progression of fruit ripening was analysed in three stages of ripening (unripe, mid ripe and ripe). The genes analysed were picked from ethylene biosynthetic pathway [SAM synthase (c27773), ACC synthase (c18584), ACC oxidase (c22984)], ethylene signalling pathway [ethylene receptor; ETR (c29681), EIN3 isoforms, (c23448_g1_i1 and c23448_g2_i1) and cell wall modifying protiens [endo beta 1–4 glucanase (c27942), expansin (c25255), pectate lyase (c27147) and polygalacturonase (c26690). All the genes showed similar trends of transcript pattern in unripe and mid ripe stage as obtained from the transcriptome data. Interestingly all these genes were highly up-regulated in ripe stage (Fig. 8).

## Conclusion

The comparative transcriptome of “Dashehari” pulp gives global landscape of differentially expressed genes in an Indian variety. The expression data obtained by RNA-seq was validated with the data obtained by qRT-PCR and also with studies carried out earlier on softening related genes. The number of genes and expression patterns of some of the genes was different from “Kent” variety and, therefore, suggests varietal differences in ripening and aroma. These transcriptome studies could be extended to other varieties to develop variety specific functional markers for each trait for ripening parameters, aroma/volatile biosynthesis enzymes and flowering time etc for use in marker assisted breeding programmes.

## Methods

### Plant material

Mango fruit of “Dashehari” variety were harvested at the mature green stage from the orchard located near Malihabad region in Lucknow India. After harvesting, unripe and mid ripe stages of fruit were used for illumina sequencing while unripe, mid ripe and ripe stages were used for 454 pyro-sequencing. The stages were defined based on fruit firmness^7^. Mangoes of different stages were photographed and cut samples were immediately frozen in liquid nitrogen and stored at −70 °C for further use. Fruit firmness was measured using a Penetrometer (Fruit Pressure Tester model FT 011).

### RNA preparation

Total RNA was extracted from mango pulp from inner zones (roughly 1 cm from the stone) of unripe, mid-ripe and ripe stages (D0I, D3I, D6I) according to the protocol of Asif *et al.*^37^. In order to remove the contaminating genomic DNA, the RNA preparation was treated with RNase free DNase I (RFD). Qualitative and quantitative estimation of the RNA was performed using Nano-Drop ND-1000 UV-Vis spectrophotometer and by 1.2% agarose gel electrophoresis.

### Transcriptome sequencing from ripe and unripe tissue

ds-cDNA was prepared using random primers with the help of SuperScript Double-Stranded cDNA Synthesis kit (Invitrogen), as per the protocol supplied with the kit. This ds cDNA was checked both quantitatively and qualitatively on Agilent 2100 Bioanalyzer DNA chip (Agilent Technologies Inc., Santa Clara, CA). The fragments smaller than 300 bp were removed using AMPure purification kit (Beckman Coulter; Agencourt, USA). The purified samples were again checked on DNA chip (Agilent 2100 Bioanalyzer, USA). RL Adapters were ligated to the fragments and these ligated products were purified as per the guidelines provided by Roche, USA. These adapter ligated fragments were then used for the clonal amplification by emulsion PCR for sequencing (following the instructions given by the manufacturers). The sequencing of libraries prepared using samples of unripe, midripe and ripe stages (postharvest) was carried out on 454-GS-FLX sequencing platform according to the instructions provided with GS FLX Titanium Kit. The 454 pyrosequenced reads of all the libraries were assembled with ROCHE GS-Assembler (version 2.5.3) with 40 base pair overlap and 96% identity for different libraries forming contigs and singletons. This data was in SFF file format.

RFD treated RNA from inner zones from unripe and mid ripe stages (D0I and D3I) of “Dashehari” was sent for the Illumina sequencing along with the SFF files obtained from 454 pyrosequencing platform. Paired-end *De-novo* transcriptome assembly was performed using Illumina HiSeq (Quality score >Q20, SciGenome, India). For this the total RNA integrity was checked using an Agilent Technologies 2100 Bioanalyzer (RIN values 8 or more were considered), followed by purification and fragmentation of mRNA using magnetic beads having poly T oligos. The next step was the synthesis of first strand and second strand cDNA synthesis respectively. Double stranded cDNA (ds cDNA) was separated and overhangs were converted into blunt ends using end repair mix (supplied with the kit). To these Adenylation was done (attachment of single A) followed by ligation of multiple indexing adapters in order to prepare them for hybridization on the flow cell. Quality control of the library was carried out using Real Time PCR and Agilent 2100 Bioanalyzer followed by sequencing using Illumina HiSeq2000 (San Diago, CA, USA).

### *De-novo* assembly of transcriptome and annotation

Using sff_extract tool SFF files (452845 reads) were extracted and then tag sequences were removed from 454 data using tag cleaner. Further quality trimming was done followed by hybrid assembly. Using Bowtie program the trimmed reads were aligned to get the assembled transcriptome. Up to 1-mismatch was allowed in the seed region (length = 31 bp). The assembled transcript was annotated using CANoPI (Contig Annotator Pipeline) for *de novo* transcriptome assembly. This pipeline performs annotation to the NCBI NR database, GO annotation and pathway annotation. Differential gene expression analysis was performed using DESeq program in R-Bioc. p value was adjusted with the Benjamini-Hochberg procedure [which controls false discovery rate (FDR)]^38^. The transcripts with p-value < 0.05, FDR <0.05 and Fold change <−2 or >2 were selected. All the annotated TAIR10 ids were used to extract the KO ids from the KAAS database (http://www.genome.jp/tools/kaas/). These Ko ids were submitted in Kegg (http://www.genome.jp/kegg/) to extract all the Metabolic pathways^39^. Also, the Uniprot ids were input in KOBAS 2.0 to retrieve the significant pathways. Heat Map was generated using the FPKM values from MeV 4.2.1.

### Pathway and GO enrichment analysis

All the GO terms were extracted from the up and down regulated genes. The GO enrichment was performed by TopGO analysis in R package and the classic fisher test analysis was used for the statistics^40^. The pathway enrichment was carried out using the Aracyc database. The statistical test was fisher exact test for p-value and Benjamini Hochberg for FDR correction.

### Comparison with NCBI database

The assembled transcripts were compared with NCBI non-redundant protein database using Blastx program. Transcripts showing matches with E-value ≤ 10^−50^ and similarity score ≥40% were retained for further annotation. Close to 90% of the assembled transcripts found using Blastx have similarity of more than 60% at protein level with the existing proteins at NCBI database. The predicted proteins from Blastx were annotated against NCBI, UniProt.

### Comparison of “Dashehari” and “Kent data”

Total transcripts obtained in our study were compared with the assembled data of Kent variety of 1D and 10 D using blastn programme. The threshold e-value was set at e^−10^ and 70% aligned length.

### Validation of differentially expressed genes by real-time PCR (qRT-PCR) analysis

Expression of randomly selected differentially expressed cell-wall hydrolases, ethylene biosynthesis and signaling components were investigated using Real-Time PCR Detection System (ABI 7500, Applied Biosystems, USA) and fast SYBR Green PCR Master Mix (VeriQuest SYBR Green qPCR Master Mix, Affymetrix) to validate 454-Illumina hybrid assembly data. cDNA samples were prepared from RFD (RNase free DNase) treated RNA with the help of RevertAid H minus M-MuLV Reverse transcriptase. Each PCR reaction was set up in total 20 μl volume containing 2X Fast SYBR green master mix, cDNA and gene-specific primers (Supplementary Table S5). MiActin was used as the reference gene. The PCR cycling conditions were as follows: 50 °C for 20 sec, 95 °C for 10 min for holding and 40 cycles of 95 °C for 15 sec and 60 °C for 1 min. The Real-time data was analysed using the ΔΔCT method^41^,^42^. All the experiments were carried out using three biological replicates along with technical replicates and the statistical analysis (standard deviation) was also performed for the data.

## Additional Information

**How to cite this article**: Srivastava, S. *et al.* Comparative transcriptome analysis of unripe and mid-ripe fruit of *Mangifera indica (var*. “Dashehari”) unravels ripening associated genes. *Sci. Rep.* **6**, 32557; doi: 10.1038/srep32557 (2016).

## Supplementary Material

Supplementary Information

## Figures and Tables

**Table 1:** Summary of reads generated and mapping on the mango transcriptome.

	Raw reads 454 (GC%)	Trimmed reads 454 (GC%)	Raw reads illimina (GC%)	Trimmed reads illimina (GC%)	No of paired end reads aligned	No of transcripts with FPKM ≥1
Un ripe inner zone	114,754 (49)	114,591 (49)	10051330 (45.74)	10020735 (45)	3227103 (64.46%)	38010
Mid ripe inner zone	140,078 (51)	139,782 (52)	14288168 (45.18)	13133966 (44.33)	4281603 (63.90%)	36302

**Table 2:** A comparative representation of different mango transcriptomes carried out so far along with “Dashehari” transcriptome.

Mango variety taken	Tissue used for sequencing	Total transcripts obtained	Average size of transcripts	Number of unigenes obtained
*Langra*	leaf	85651	536	30509
*Zill*	Pericarp and peel	124002	838	54207
*Kent*	mesocarp	80969	836	52948
*Dashehari*	Inner zone of mesocarp	71733	942	44472

**Table 3:** Functional annotation of *M. indica var*.

Down- regulated			
	Term	Annotated	P-value
Biological Processes	GO:0010200	response to chitin	4.40E-10
	GO:0009753	Response to jasmonic acid	1.10E-09
	GO:0009651	Response to salt stress	6.60E-08
	GO:0009414	Response to water deprivation	1.10E-08
	GO:009611	Response to wounding	2.70E-08
	GO:0046686	Response to cadmium ion	4.30E-08
	GO:0042742	Defense response to bacterium	2.00E-07
	GO:0080167	Response to karrikin	8.30E-07
	GO:0006857	Oligopetide transport	1.80E-06
Molecular functions	GO:0009409	Response to cold	4.10E-06
	GO:0016168	Chlorophyll binding	8.70E-07
	GO:0004590	Orotidine-5′-phosphate decarboxylate activity	1.40E-05
	GO:0043295	Glutathione binding	2.60E-05
	GO:0020037	Heme binding	5.80E-05
	GO:0005507	Copper ion binding	0.00013
	GO:0004096	Catalase activity	0.00032
	GO:0030599	Pectinesteraseactivity	0.00035
	GO:0015035	Protein disulphide oxidoreductase activity	0.00052
	GO:0008810	Cellulase activity	0.00068
Up-regulated			
Biological Processes	Term	Annotated	P-value
	GO:0045490	Pectin catabolic process	5.00E-09
	GO:0006885	Regulation of pH	2.40E-06
	GO:0009664	Plant type cell wall organization	2.70E-06
	GO:0006754	ATP biosynthetic process	6.70E-06
	GO:0055085	Transmembrane transport	7.90E-06
	GO:0006562	Proline catabolic process	0.00031
	GO:0006788	Heme oxidation	0.00031
	GO:0009415	Response to water	0.00031
	GO:0006557	S-adenosylmethioninamine biosynthesis	0.0006
	GO:0006470	Protein dephosphorylation	0.00069
Molecular functions	GO:0030570	Pectate lyase activity	1.60E-11
	GO:0022891	Substrate specific transmembrane transport activity	2.50E-06
	GO:0015385	Sodium:proton antiporter activity	2.30E-05
	GO:0003700	Sequence specific DNA binding transcription factor	3.70E-05
	GO:0034450	Ubiquitin-ubiquitin ligase activity	4.80E-05
	GO:0004176	ATP-dependent peptidase activity	5.20E-05
	GO:0004722	Protein serine/threonine phosphatase activity	6.00E-05
	GO:0005215	Transporter activity	7.00E-05
	GO:0004315	3-oxoacyl-[acyl-carrier-protein] synthase activity	1.00E-04
	GO:0004657	Proline dehydrogenase activity	1.00E-04

“Dashehari” *transcripts: Up and down regulated GOSlim terms in M. indica* transcripts under biological process (BP) and molecular function categories (MF).
