# Supplementary material for: Comparative transcriptome analysis of unripe and mid-ripe fruit of Mangifera indica (var. “Dashehari”) unravels ripening associated genes
Source: Sci Rep. 2016 Sep 2;6:32557. doi: 10.1038/srep32557 (PMC5009307; doi:10.1038/srep32557)
Supplement: Supplementary Information [file srep32557-s1.pdf]

Supplementary information

Comparative transcriptome analysis of unripe and mid-ripe fruit of *Mangifera indica* (*var.Dashehari*) unravels ripening associated genes

Smriti Srivastava[1,2], Rajesh K. Singh[3], Garima Pathak[1,2], Ridhi Goel[1,2], Mehar Hasan Asif[1], Aniruddha P Sane[1], Vidhu A Sane[1*]

[1] CSIR- National Botanical Research Institute, Lucknow-226001, India. [2]Academy of Scientific and Innovative Research (AcSIR), Anusandhan Bhawan, 2 Rafi Marg, NewDelhi-110001. [3] Umeå Plant Science Centre, Umea, Sweden

* Corresponding author. Tel : 91 522 2297978; Fax: 91 522 2205836

E.mail: sanevidhu@rediffmail.com, va.sane@nbri.res.in

Running title: Transcriptome analysis of "Dashehari" mango

Figure S1 Differential gene expression of top 100 contigs. Heat map was drawn using MeV software using standard settings.

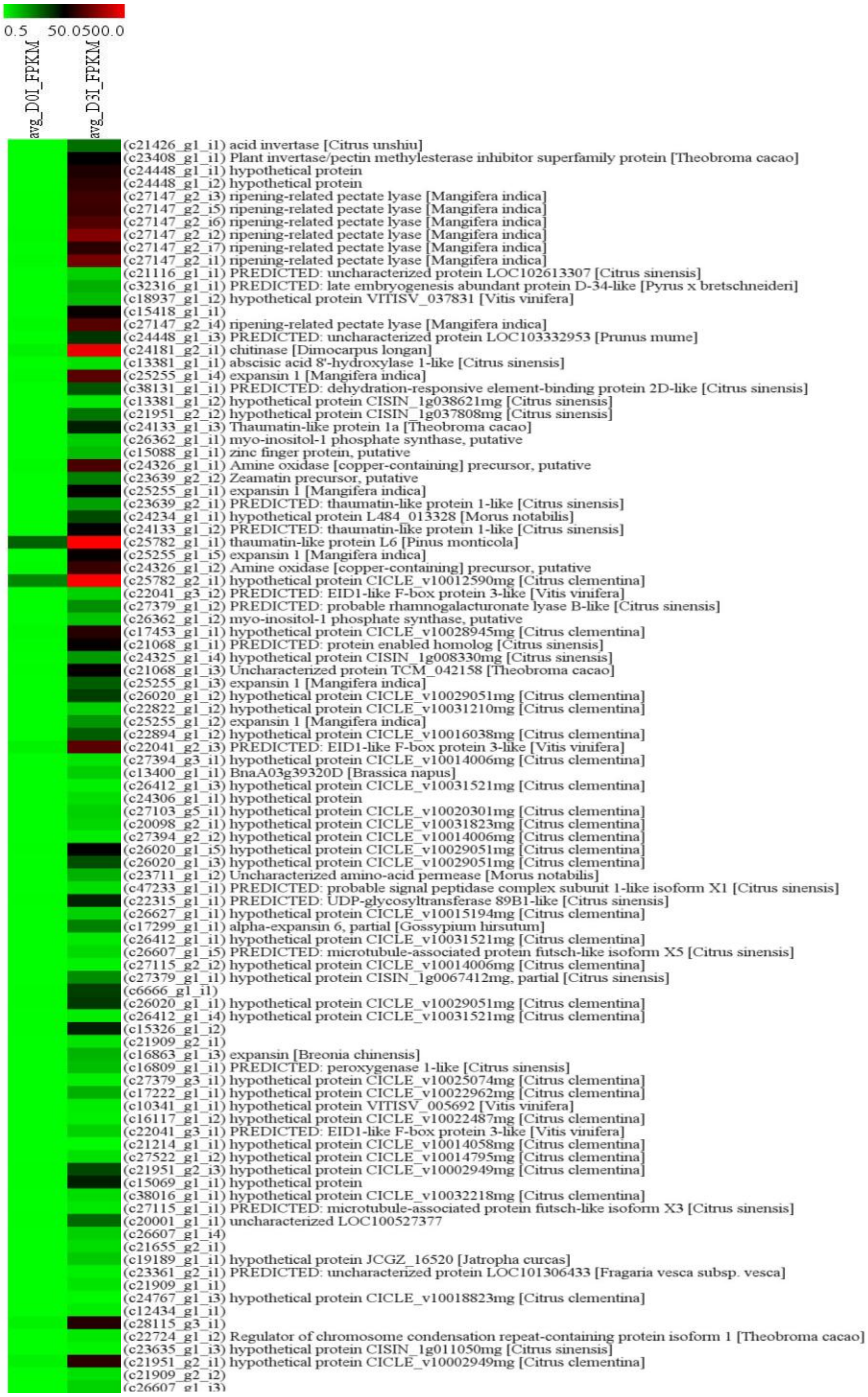

Figure S2 a: Multiple sequence alignment of different contigs representing 1-aminocyclopropane-1-carboxylic acid synthase. Sequence with green background shows the start and stop codons. Mi stands for *Mangifera indica*, Cs for *Citrus sinensis*, At for *Arabidopsis thaliana* and Tc for *Theobroma cacao*.

```
Mic48715      ------------------------------------------------------------
Mic38692      ------------------------------------------------------------
Mic32921      ------------------------------------------------------------
AtACS6        ------------------------------------------------------------
Mic50867      ------------------------------------------------------------
CsACS         ------------------------------------------------------------
Mic18584      ------------------------------------------------------------
Mic5878       ------------------------------------------------------------
TcACS10       CCCAAAATCTAAAAATCCCCCCCTTCAAGCAAGGGACGGCTCAGAAAGAATCTATATCCC
Mic22459      ------------------------------------------------------------

Mic48715      ------------------------------------------------------------
Mic38692      ------------------------------------------------------------
Mic32921      ------------------------------------------------------------
AtACS6        ------------------------------------------------------------
Mic50867      ------------------------------------------------------------
CsACS         ------------------------------------------------------------
Mic18584      ------------------------------------------------------------
TcACS10       ATAATTCCCCCCAAAAAATAAAATAAAAAAGAAAACCTCAAAACTCAGAAAACCAGAAAA
Mic22459      ------------------------------------------------------------

Mic48715      ------------------------------------------------------------
Mic38692      ------------------------------------------------------------
Mic32921      ------------------------------------------------------------
AtACS6        ------------------------------------------------------------
Mic50867      ------------------------------------------------------------
CsACS         ------------------------------------------------------------
Mic18584      ------------------------------------------------------------
TcACS10       AAAAAAAAAAAAAAAGGGAAAGAGACCACAAAGCTATTATTTTTATTCATCCGTTGAACA
Mic22459      ------------------------------------------------------------

Mic48715      ------------------------------------------------------------
Mic38692      ------------------------------------------------------------
Mic32921      ------------------------------------------------------------
AtACS6        ------------------------------------------------------------
Mic50867      ------------------------------------------------------------
CsACS         ------------------------------------------------------------
Mic18584      ------------------------------------------------------------
TcACS10       TTAATCAGAAAACCCAGAAAAACCCATATTTTCAATTCCCATTTTTCATCTTCATTTTGA
Mic22459      ------------------------------------------------------------

Mic48715      ------------------------------------------------------------
Mic38692      ------------------------------------------------------------
Mic32921      ------------------------------------------------------------
AtACS6        ------------------------------------------------------------
Mic50867      ------------------------------------------------------------
CsACS         ------------------------------------------------------------
Mic18584      ------------------------------------------------------------
TcACS10       GTTTTAGTGGCCATGACTTCACTGCAATGACGCAAACCCGATTTTCCCGGACTCGGACCC
```

```
Mic22459       ------------------------------------------------------------

Mic48715       ------------------------------------------------------------
Mic38692       ------------------------------------------------------------
Mic32921       ------------------------------------------------------------
AtACS6         ------------------------------------------------------------
Mic50867       ------------------------------------------------------------
CsACS          ------------------------------------------------------------
Mic18584       ------------------------------------------------------------
TcACS10        CCGAACCCGAAAAGGAACAAGCCAGAGGGGGAGGAGGAGCCGCCATGAGAGTGATAGTTC
Mic22459       ------------------------------------------------------------

Mic48715       ------------------------------------------------------------
Mic38692       ------------------------------------------------------------
Mic32921       ------------------------------------------------------------
AtACS6         ------------------------------------------------------------
Mic50867       ------------------------------------------------------------
CsACS          ------------------------------------------------------------
Mic18584       ------------------------------------------------------------
TcACS10        CTCTACAAGGTGTTGTTCAAGGAAGAGGAGGCTTAATCTTAGGCTCCATAATTCCTTGCG
Mic22459       ------------------------------------------------------------

Mic48715       ------------------------------------------------------------
Mic38692       ------------------------------------------------------------
Mic32921       ------------------------------------------------------------
AtACS6         ------------ATCAAACCATAACTTCCAAATCTCAACAGAACCA------AAAACAAA
Mic50867       ------------------------------------AACAGAACCA------AAAACAAA
CsACS          ------------------------------------------------------------
Mic18584       --CTCTTCTCTGTTCTCTCTTTCTCTCCTATCTATCTGCATATAT---------------
TcACS10        CGCTCTTTTACTTTCTGCAATTCTATTTGAAGCGACACCGTAATGACCAGGATGACCAGA
Mic22459       ------------------------------------------------------------

Mic48715       ------------------------------------------------------------
Mic38692       ------------------------------------------------------------
Mic32921       ------------------------------------------------------------
AtACS6         AGAAACC--TATATTAAAG-----------------------------------------
Mic50867       AGAAACC--TATATTAAAG-----------------------------------------
CsACS          ------------ATTACTT-----------------------------------------
Mic18584       ATTTGCC--ATTCGACACT-----------------------------------------
TcACS10        ACGAATCGAATTCCCCGGGTCAAAACCAGGGGACACGGTCGCCGTCGTCGGGGCAGTTGA
Mic22459       ------------------------------------------------------------

Mic48715       ------------------------------------------------------------
Mic38692       ------------------------------------------------------------
Mic32921       ------------------------------------------------------------
AtACS6         ----------------AAGAAACAAAAAATGGTGGCTTTTGCAACAGAGAAGAAGCAAGA
Mic50867       ----------------AAGAAACAAAAAATGGTGGCTTTTGCAACAGAGAAGAAGCAAGA
CsACS          ----------------------TCAGAAACAGATTAAATGGCCTTCGCGTTGAGTAACCA
Mic18584       ----------------------CTCTAAGACACAAAAATGGCGTCCATCTTTAGAAAACA
TcACS10        CTGAGCTTCCTGGGTTGACACGTAATCTGTCTCGGGCTTTGCTATCGCCAAGAAGTCCGA
Mic22459       ------------------------------------------------------------

Mic48715       ------------------------------------------------------------
Mic38692       ------------------------------------------------------------
```

```
Mic32921      ------------------------------------------------------------
AtACS6        TCTG----AATCTATTGTCTAAAATCGCCTCCGGTGACGGTCACGGCGAGAATTCCTCTT
Mic50867      TCTG----AATCTATTGTCTAAAATCGCCTCCGGTGACGGTCACGGCGAGAATTCCTCTT
CsACS         AGTG----CAATTGTTGTCCAAGATTGCAGCCGGCAATGGCCATGGTGAAGATTCTCCGT
Mic18584      AGAA----GAAATGTTGTCCAAGATTGCAATCGGTAATGGACATGGTGAAAACTCTCCAT
TcACS10       GTGGACCCGTTTCAGTCTCAGGTCGAGTTAGTGGGATTGTGAAGGCTGCCGATTCTCCTT
Mic22459      ------TTATTTCA--------------------GCTTTTAAATGGAAAAGGTTGTT--T

Mic48715      ------------------------------------------------------------
Mic38692      ------------------------------------------------------------
Mic32921      ------------------------------------------------------------
AtACS6        ATTTCGATGGTTGGAAAGCTTATGAAGAAAACCCATTTCACC------CAATTGATAGAC
Mic50867      ATTTCGATGGTTGGAAAGCTTATGAAGAAAACCCATTTCACC------CAATTGATAGAC
CsACS         ATTTCGATGGCTGGAAGGCTTTCGAAAGTGATCCTTATCATC------CCACCAAGAATC
Mic18584      ATTTTGATGGGTGGAAGGCATATGAAACTGATCCATATCATC------CCACTAAGAATC
TcACS10       ATTATGCCGGCTTAAGGCGGGTCAAGGAGGACCAGTATGATG------AATTGGGTAATC
Mic22459      TCTTTGTCAACTTATATTGTTAAAAATAAACCTGTTTTTGTAATTTCAATGTGGCTGATG

Mic48715      ------------------------------------------------------------
Mic38692      ------------------------------------------------------------
Mic32921      ------------------------------------------------------------
AtACS6        CTGACGGAGTTATTCAAATGGGTCTCGCTGAAAATCAGCTTTGTGGAGATTTGATGCGTA
Mic50867      CTGACGGAGTTATTCAAATGGGTCTCGCTGAAAATCAGCTTTGTGGAGATTTGATGCGTA
CsACS         CTAATGGGGTTATCCAGATGGGTCTAGCTGAGAACCTGCTTTGCCATGAATTGGTTGAAG
Mic18584      CCAAGGGAGTCATCCAGATGGGTTTAGCCGAAAATCTGCTTTGCTTTGATTTAGTTCAAG
TcACS10       CGAATGGGGTCATCCAGCTGGGTTTAGCGGAAAACAAGTTGTCGTTGGATTTGGTTAAGA
Mic22459      AGTTTGAGCATTTGCTATGTTATGTTTGGTTGACGTAGTTGTCATTAGATTTGGTTGAGG

Mic48715      ------------------------------------------------------------
Mic38692      ------------------------------------------------------------
Mic32921      ------------------------------------------------------------
AtACS6        AATGGGTTTTAAAACATCCAGAAGCTTCGATTTGT---------ACATCAGAAGGTGTGA
Mic50867      AATGGGTTTTAAAACATCCAGAAGCTTCGATTTGT---------ACATCAGAAGGTGTGA
CsACS         ACTGGCTCATGAACAACCCAAAGGCCTCCATTTGC---------TCAGCTGAAGGCCTAA
Mic18584      AATGGGTCTTAAGCAACCCAGAAGCCTCTATCTGC---------ACTGCCGAAGGTATAA
TcACS10       ACTGGCTAGCAGAGAATGCGAGGGAAGCGATCTTG---------GGAAATGGAAAGGAGC
Mic22459      ATTGGCTTGACCAGAATGCAAGAGAGGCGTTAGTGGGTGGAGGAGCAAATTGTGAGCGAT

Mic48715      ------------------------------------------------------------
Mic38692      ------------------------------------------------------------
Mic32921      ------------------------------------------------------------
AtACS6        ATCAATTCAGTGACATTGCCATTTTTCAAGATTATCATGGCTTGCCTGAATTCAGACAAG
Mic50867      ATCAATTCAGTGACATTGCCATTTTTCAAGATTATCATGGCTTGCCTGAATTCAGACAAG
CsACS         ATGAATTCAAGGGTATAGCTATCTTTCAGGATTATCACGGCTTGCCAGAGTTCAGAAATG
Mic18584      GTGATTTCAGAGATATCGCTATCTTTCAGGATTATCACGGCTTGCCAGAGTTCAGAAATG
TcACS10       TGAGCATTAGCGGGATTGCAACTTACCAGCCTTTTGATGGATTGATGGAGTTCAAAGTGG
Mic22459      CAAATAGAAAGGGGATTGCCAGTTACCATGCTTTTGATGGACTGATGGAGTTGAAAGTGG

Mic48715      ------------------------------------------------------------
Mic38692      ------------------------------------------------------------
Mic32921      ------------------------------------------------------------
AtACS6        CTGTAGCGAAATTTATGGAGAAGACTAGAAATAACAAAGTTAAGTTTGATCCTGACCGGA
Mic50867      CTGTAGCGAAATTTATGGAGAAGACTAGAAATAACAAAGTTAAG----------------
CsACS         CTGTTGCAAAATTTATGGGGAAAGTGAGAGGGAACAGAATTACATTCGATCCCGACCGTA
Mic18584      CTGTTGCAAATTTTATGGCAAGAGTGAGAGGGAATAGAGTCAAATACGACCCTGATCGAA
```

```
TcACS10       CTGTGGCAGGATTCATGGCTCAAGTCATGGAGAAAGCTGTTTCCTTTAATCCTTCGCAGA
Mic22459      CTGTTGCTGGGTTTATGTCCCAAGTCATGGAAAAAGAAATTTCCTTCAATCCTTCACAAA

Mic48715      ------------------------------------------------------------
Mic38692      ------------------------------------------------------------
Mic32921      ------------------------------------------------------------
AtACS6        TTGTTATGAGCGGCGGCGCAACCGGAGCACACGAGACGGTTGCTTTCTGTTTAGCTAATC
Mic50867      ------------------------------------------------------------
CsACS         TTGTTATGAGTGGTGGAGCAACCGGAGCTCATGAGACGGTTGCTTTCTGCTTAGCCGACC
Mic18584      TTGTTATGAGCGGTGGAGCAACCGGAGCACATGAGACGGTTGCCTTTTGTTTGGCTGATC
Mic5878       ------------------------------------------------------------
TcACS10       TAGTGCTGACTGCTGGTGCGACCCCTGCAATCGAGATTCTTAGCTTCTGCTTAGCAGATG
Mic22459      TAGTATTAACAGCTGGTGCATCTCCTGCAGTAGAGATACTCAGCTTCTGCCTTGCAGACA

Mic48715      ------------------------------TTGGTTTCAGGTTTGATAGAGATTTGAGAT
Mic38692      ------------------------------------------------------------
Mic32921      ------------------------------------------------------------
AtACS6        CCGGCGATGGTTTCTTAGTTCCAACCCCTTATTATCCAGGGTTTGATAGAGATTTGAGAT
Mic50867      ------------------------------------------------------------
CsACS         CCGGCGACGCATTCTTAGTACCCACTCCCTATTATCCAGGATTTGATCGAGATTTGAGAT
Mic18584      CCGGTGAAGCATTTTTGGTGCCCACTCCTTACTATCCAGGATTTGATCGAGATTTGAGAT
TcACS10       CTGGAAATGCATTTCTTGTTCCAACACCATATTACCCTGGTTTTGACAGGGATGTAAAAT
Mic22459      GCGGGAATGCATTTCTTGTTCCAACACCTTATCATCCTAGTCTTGATAGGGATGTAAAAT

Mic48715      GGAGAACCGGAGTGAATCTTGTACCGGTTACTTGTCATAGCTCTAATGGGTTCAAGATTA
Mic38692      ------------------------------------------------------------
Mic32921      ------------------------------------------------------------
AtACS6        GGAGAACCGGAGTGAATCTTGTACCGGTTACTTGTCATAGCTCTAATGGGTTCAAGATTA
Mic50867      ------------------------------------------------------------
CsACS         GGAGAACAGGAGTTCAACTTGTTCCAGTTGTATGTGACAGCTCTAACGATTTCAAGGTCA
Mic18584      GGAGAACAGGAGTTCAACTTTTTCCAGTTGTGTGTGACAGTTCTAACAATTTCAAGATTA
TcACS10       GGAGAACTGGGGTGGAGATAATACATGTTCCTTGCCGAAGTGCTGACAACTTCAATTTGA
Mic22459      GGCGAACTGGAGTAGACATAATCCCTGTTCCCTGCCGCAGTGCTGACAACTTCAGTGTAA

Mic48715      CGGTGGAAGCCTTGGAAGCTGCTTACGAAAACGCGAGAAAATCGAATATTCCGGTTAAGG
Mic38692      ------------------------------------------------------------
Mic32921      ------------------------------------------------------------
AtACS6        CGGTGGAAGCCTTGGAAGCTGCTTACGAAAACGCGAGAAAATCGAATATTCCGGTTAAGG
Mic50867      ------------------------------------------------------------
CsACS         CCATGGAAGCCTTAGAAGCGGCTTATGAAAAAGCCCAAGAATCCAACATCAGAATAAAGG
Mic18584      CAAGAGAAGCCGTGGAAGCAGCATATGAAAAAGCTCAAGAAGACCACATCAGAATCAAGG
TcACS10       GTATAACTGCTCTCGATATAGCATTCAACCAGGCAAAGAAACGTGGGCTGAAAGTGCGTG
Mic22459      GTATTACTGCTCTAGATCGAGCTTTCAACCAGGCTAGGAAGCGTGGTATCAGAGTTCGTG

Mic48715      GTTTACTTGTAACCAATCCTTCAAACCCGCTTGGTACGACGTTAGACCGGGAATGTTTGA
Mic38692      ------------------------------------------------------------
Mic32921      ------------------------------------------------------------
AtACS6        GTTTACTTGTAACCAATCCTTCAAACCCGCTTGGTACGACGTTAGACCGGGAATGTTTGA
Mic50867      ------------------------------------------------------------
CsACS         GTGTCCTTATTACCAATCCATCAAACCCATTGGGCACTTTCTTAGACCGAGAAACCCTAA
Mic18584      GTTTGCTCCTCACAAATCCATCGAACCCGCTGGGGACTTGTTTGGACAGAGAAACACTAA
TcACS10       GGATCATAATATCAAATCCATCAAATCCTGTTGGGAATCTTCTAAGTCGGGAAACACTTT
Mic22459      GTATTATAATATCAAATCCTGCAAGTCCTGTTGGCAATCTATTTAACCGGGATACACTTT
```

```
Mic48715      AGTCTCTTGTTAACTTCACTAATGACAAAGGGATTCATCTTATTGCTGATGAGATTTATG
Mic38692      ------------------------------------------------------------
Mic32921      ------------------------------------------------------------
AtACS6        AGTCTCTTGTTAACTTCACTAATGACAAAGGGATTCATCTTATTGCTGATGAGATTTATG
Mic50867      ------------------------------------------------------------
CsACS         AAGATATAGTGAGCTTCATCAATGAAAAGAACATCCATTTAGTCTGTGATGAGATTTATG
Mic18584      GAAGTTTAGTAAGCTTCATTAATGAAAAGAACATCCACTTAGTCTGCGACGAGATTTATG
TcACS10       ATAACCTTTTAGACTTTGCCAGAGAGAAGAACATCCATATTGTCTCTAATGAAATATTGG
Mic22459      ACAGCCTTCTGGACTTTGCTAGGGAGAAGAAGATCCATATAGTCTCTAACGAATTGCTAG

Mic48715      CTGCTACTACTTTTGG---TCAATCCGA--------------------------------
Mic38692      ------------------------------------------------------------
Mic32921      ------------------------------------------------------------
AtACS6        CTGCTACTACTTTTGG---TCAATCCGAGTTCATAAGTGTTGCGGAAGTAATCGAGGAGA
Mic50867      ------------------------------------------------------------
CsACS         CTGCCACAGTGTTCACCAAGGAGCCTAGTTTCGTTAGCATCGCGGAGATAATAGACCAAG
Mic18584      CTGCCACAATCTTCATGGGCCAGCCCGATTTCATTAGCATCTCTGAAATTATAGAAGAAG
TcACS10       CTGGATGTACTCATGGAAATGAA---GAATTTGTGAGCATGGCTGAAATTGTTGACCTGG
Mic22459      TTGGGTCCACTTATGGAAGTGAA---GAATTTGTGAGCATGGCAGATATCATTGATGTAG

Mic48715      ------------------------------------------------------------
Mic38692      ------------------------------------------------------------
Mic32921      -----------------------------------------------------------C
AtACS6        TCGAAGATTGTAACCGCGATTTGATACATATTGTGTATAGTCTATCTAAAGATATGGGTC
Mic50867      ------------------------------------------------------------
CsACS         ACATTGCATGCAATCGCAATCTCATTCACATTGTATACAGTCTTTCTAAGGACATGGGAT
Mic18584      ATATTCACTGCAATCGCAATCTCATCCACCTTGTTTACAGTCTTTCAAAGGATCTGGGGT
TcACS10       ---AAGATGTTGACCGGAAAAGAGTCCATCTAGTATACGGTTTGTCAAAAGATCTTTCTC
Mic22459      ---AAGACCTTGACAGGGACAGAGTTCATATAGTATATGCTCTATCAGAAGACCTTTCTC

Mic48715      ------------------------------------------------------------
Mic38692      ------------------------------------------------------------
Mic32921      TGCCTGGTTTAAGAGTTGGTATAGTATACTCTTACAATGACAGGGTGGTTCAGATCGCAA
AtACS6        TGCCTGGTTTAAGAGTTGGTATAGTATACTCTTACAATGACAGGGTGGTTCAGATCGCAA
Mic50867      ------------------------------------------------------------
CsACS         TCCCTGGCTTTAGGGTCGGTATCATTTACTCGTACAATGATCAAGTAGTGAGCTGTGCTC
Mic18584      TCCCAGGCTTTAGGGTCGGCATTATATACTCATACAACGATACAGTTGTGAGTTGCGCCC
TcACS10       TGCCAGGCTTTAGGGTTGGTGTTATTTACTCTTTCAATGAGGAGGTTCTGGCTGCTGCGA
Mic22459      TTCCAGGTTTTAGGATGGGAATTATTTACTCCTACAATGAGAATGTTCTGTCCGCTACTA

Mic48715      ------------------------------------------------------------
Mic38692      ------------------------------------------------------------
Mic32921      GGAAAATGTCGAGTTTCGGTCTTGTTTCGTCACAAACGCAGCATTTGATCGCTAAAATGT
AtACS6        GGAAAATGTCGAGTTTCGGTCTTGTTTCGTCACAAACGCAGCATTTGATCGCTAAAATGT
Mic50867      ------------------------------------------------------------
CsACS         GCAAAATGTCTAGCTTTGGACTTGTATCCTCACAAACCCAACATTTAATCGCAACAATGT
Mic18584      GCAAAATGTCAAGCTTTGGACTTGTATCATCACAAACTCAACATTTAATCGCTTCAATGT
TcACS10       AAAAGTTGACGAGGTTCTCATCTATTTCAGCTCCAACCCAGCGTCTGCTCATCTCTATGC
Mic22459      GAAAAATGGCTAGGTTCTCATCTGTTTCAGCTCCAATTCAGCAATTGCTAGTTTCAATGC

Mic48715      ------------------------------------------------------------
Mic38692      ------------------------------------------------------------
Mic32921      TATCCGATGAAGAGTTTGTAGACGAGTTTATCCGCGAGAGCAAATTGCGGTTAGCTGCAA
AtACS6        TATCCGATGAAGAGTTTGTAGACGAGTTTATCCGCGAGAGCAAATTGCGGTTAGCTGCAA
Mic50867      ------------------------------------------------------------
```

```
CsACS          TATCGGATGATCAGTTTGTGGAGAAGTTCATCGCCGAGAGTGCTAAAAGAATAGCAGAAA
Mic18584       TATCAGATGATGAATTTGTGGATAGGTTCATTACTGAGAGTGCTAAAAGGCTTGCAAAAA
TcACS10        TATCCGATACAAAATTTGTTCAAACATTTATTACCATTAACAGGGAGAGGCTTCATAGAA
Mic22459       TTACCGACACTAAATTTGTTGACAAGTTCGTCAAGACTAATAGGGAGAGACTTGGAAGAA


Mic48715       ------------------------------------------------------------
Mic38692       ------------------------------------------------------------
Mic32921       GGCACGCTGAGATAACCACCGGTTTAGATGGTTTAGGGATTGGTTGGTTAAAGGCCAAAG
AtACS6         GGCACGCTGAGATAACCACCGGTTTAGATGGTTTAGGGATTGGTTGGTTAAAGGCCAAAG
Mic50867       ------------------------------------------------------------
CsACS          GGCATAAGGCATTCACTTGGGGACTTTCCCAAGTGGGCATTGGGTGTTTAAAGAGCAATG
Mic18584       GGCACAGAGCCTTCACATGGGGGCTATCTCAAGTAGGCATTGGTTGTTTGAAGAGCAATG
TcACS10        TGTATGTTCAGTTCGTGGAAGGGTTGAAAAAATTGGGCATAGAGTGCATAAAGAGTAGTG
Mic22459       TGTATGTTGAGTTTGCAGGAGGATTAAAACAGTTGGGCATTGAGTGCACAAAAAGTAGTG


Mic48715       ------------------------------------------------------------
Mic38692       -----------------------------------------ACAGCAACGTTTGATTCGG
Mic32921       CCGGTTTGTTCTTGTGGATGGATTTAAGAAATCTTTTGAAGACAGCAACGTTTGATTCGG
AtACS6         CCGGTTTGTTCTTGTGGATGGATTTAAGAAATCTTTTGAAGACAGCAACGTTTGATTCGG
Mic50867       ------------------------------------------------------------
CsACS          CTGGCCTGTTCCTATGGATGGATTTGCATCATCTTCTGAAGGAGCAAACATACGAAGCTG
Mic18584       CGGGGCTATTTTTCTGGATGGATTTGCATCATCTCCTCAAGGAGCAAACTGATGAAGCAG
TcACS10        GGGGTTTCTACTGTTGGGCTGATATGAGCGGGTTAATAAGCTCTTATAGTGAGAAAGGGG
Mic22459       GGGGTTTCTACTGTTGGGCTGACATGAGTGGATTAATCAGCTCTTACAGTGAGAAAGGGG


Mic48715       ------------------------------------------------------------
Mic38692       AAACCGAACTATGGCGTGTGATTGTTCACCAAGTGAAGCTCAACGTGTCTCCAGGCGGTT
Mic32921       AA----------------------------------------------------------
AtACS6         AAACCGAACTATGGCGTGTGATTGTTCACCAAGTGAAGCTCAACGTGTCTCCAGGCGGTT
Mic50867       ------------------------------------------------------------
CsACS          AGATGGCGTTATGGCGAGTGATAATTAACGAAGTCAAGCTTAATGTTTCTCCTGGATCTT
Mic18584       AGATAGAACTGTGGAAAGTGATAATCAACGAAGTTAAATTAAATGTTTCTCCGGGTTCTT
TcACS10        AACTTGAGCTTTGGGATAAGTTGTTGAATATAGCCAAGGTCAATGTAACTCCAGGGTCTT
Mic22459       AGCTTGAACTTTGGGATAAGTTGTTGAATGTGGCTAAGCTCAATGTAATTCCTGGATCTT


Mic48715       ------------------------------------------------------------
Mic38692       CGTTCCATTGCCATGAACCGGGATGGTTTAGAGTATGTTTTGCGAATATGGACCATAAGA
Mic32921       ------------------------------------------------------------
AtACS6         CGTTCCATTGCCATGAACCGGGATGGTTTAGAGTATGTTTTGCGAATATGGACCATAAGA
Mic50867       ------------------------------------------------------------
CsACS          CTTTTCACTGCCCGAATCCAGGGTGGTTTAGAGTTTGCTTCGCCAACATGGATGATCGGA
Mic18584       CCTTTCATTGCGCTAATCCAGGATGGTTTCGGGTTTGTTTTGCCAACATGGATGATGAAA
TcACS10        GTTGTCATTGTATTGAACCTGGATGGTTCCGCTTCTGTTTTGCCACATTGACAGAGAAAG
Mic22459       CTTGTCACTGTATTGAACCAGGATGGTTCTGTTTCTGTCTTACTTTGTTGACTGAAAAAG


Mic48715       ------------------------------------------------------------
Mic38692       CGATGGAGACAGCTCTAGAGAGGATTAGAGTGTTCACTAGCCAACTTGAGGAGGAGACTA
Mic32921       ------------------------------------------------------------
AtACS6         CGATGGAGACAGCTCTAGAGAGGATTAGAGTGTTCACTAGCCAACTTGAGGAGGAGACTA
Mic50867       ------------------------------------------------------------
CsACS          CGATGGAAATTGCTCTATCAAGAATTACAAGCTTTATGCTTAAGAATGTGGAAGCCAAGG
Mic18584       CCACGGAAGTTGCTTTAGCAAGAATCAGAAGTTTTATGCGTAAGAATAAGGAAGCGAAGA
TcACS10        ATATCCCTGTGGTTATGGAACGGATTCAGAAAGTTTCTGAAACCTGTAAATTGAATAGAT
Mic22459       ATATCCCTGTAGTTATGGAACGAATTCTAAGAATTTCCAAAACCTGTAAAATTCATACTT
```

```
Mic48715      ------------------------------------------------------------
Mic38692      AACCGATGGCTGCAACAA----CTATGATGGCTAAAAAGAAGAAGAAGTGTTGGCAGAGT
Mic32921      ------------------------------------------------------------
AtACS6        AACCGATGGCTGCAACAA----CTATGATGGCTAAAAAGAAGAAGAAGTGTTGGCAGAGT
Mic50867      ------------------------------------------------------------
CsACS         TGCCTAAT----------------------------AAGAAGAAACTTTGCTGGCAAAGA
Mic18584      TGCCTGCG-------------------------------AAGAAATTGTGCAGGCAAAGA
TcACS10       GAAATGTTGTTTGTTTATTCTATGGTTTTAGAC---AACCCGAGATTTTTTTAT------
Mic22459      GAAGTGCAATTGGATTATTATT--------------------------------------

Mic48715      ------------------------------------------------------------
Mic38692      AACCTCAGGTTAAGCTTTAGTGACACGAGGCG--GTTCGATGATGGCTTCTTCTCGCCTC
Mic32921      ------------------------------------------------------------
AtACS6        AACCTCAGGTTAAGCTTTAGTGACACGAGGCG--GTTCGATGATGGCTTCTTCTCGCCTC
Mic50867      ------------------------------------------------------------
CsACS         AGCCTTCGGCTCAGCATGTCATCTCGAAGGATGGATGACTTCATGGCTTCACCATGCATG
Mic18584      AGTCTCAGGCTCAGCACTTCATTCAGGAGATTGGATGATATCATGAACTCGCCATGCATG
TcACS10       ------TGAAAGCTCTTTTCAGTTGACACTAA--AGCAGACAGTAGTAGTCCCTGGCCTG
Mic22459      ------------------------------------------------------------

Mic48715      ------------------------------------------------------------
Mic38692      ATTCGCCTG--------------TGCCGCCT-----------------------------
Mic32921      ------------------------------------------------------------
AtACS6        ATTCGCCTG--------------TGCCGCCTT---CTCCGCTAGTCCGTGCACA-GACTT
Mic50867      ------------------------------------------------------------
CsACS         TCTCC----------------------TCAAT---CACCTCTTGTTCAGGCCCG-GACTT
Mic18584      TCTCC----------------------TAATT---CACCTCTTGTTCGAACCAG-AACTT
TcACS10       GTTCAAGAACCAATCAATGCCATTAAGACCTTAATTTTCATATGTAGGGATCGGTGCAAA
Mic22459      ------------------------------------------------------------

Mic48715      ------------------------------------------------------------
Mic38692      ------------------------------------------------------------
Mic32921      ------------------------------------------------------------
AtACS6        AAGACCGTCTCATA------------TTTTGACTAGACCAGT------------------
Mic50867      ------------------------------------------------------------
CsACS         AAGTGCAATAATAATTGGAGCTACACGTTCGTATGACCAAGTCAAGTGTAATTGAGGCAA
Mic18584      AATCAATTAGGTGCAATAATCTCTGCACCAAGCTCAT------------AATTGATTAAG
TcACS10       TCACAAGCTATTGCATAGGGTTAAACTGTTTTGGGATCTGTACGCACAAAAAGCTTGGAA
Mic22459      ------------------------------------------------------------

Mic48715      ------------------------------------------------------------
Mic38692      ------------------------------------------------------------
Mic32921      ------------------------------------------------------------
AtACS6        -CGTCGTTAA-TTAAAAAGTCAATTCTTT---------AGATTGATTTT-GACACATTTA
Mic50867      ------------------------------------------------------------
CsACS         TTT-AGTTCAATTATTTTTTTCATTTTGTTCGTAACTAAGAGAAACAAT-GGCTCATGCA
Mic18584      TTCTGGTTCAAACAAGAGGTGAATTAGGA----------------GACT-GCGCCAAGCT
TcACS10       ATCTGATTGTCTCATGTTGAGCAATTGGATCAGAAGCTTGATTAGTATAAAGATACTGGA
Mic22459      ------------------------------------------------------------

Mic48715      ------------------------------------------------------------
Mic38692      ------------------------------------------------------------
Mic32921      ------------------------------------------------------------
AtACS6        TCTGATTAAATCAAATGTATAGCTACGACTATCAAGTTGATTTTTTCTTTCTTTTA----
```

```
Mic50867      ------------------------------------------------------------
CsACS         TTTTTTTTTCCTCCCTGTGTGGAGTCGACCAGAATTGTAGTCCTTCGTTTGG-GAT----
Mic18584      CATAATATACCGTCAAGTGTAAAGAC--TTATGATTGACGCATTTATTTTGT--------
TcACS10       TCTT----GTGAAACTGAATAAAATAGAAAAGGTGTGTTGAATTTGGTGCATCAGTGTGT
Mic22459      ------------------------------------------------------------

Mic48715      ------------------------------------------------------------
Mic38692      ------------------------------------------------------------
Mic32921      ------------------------------------------------------------
AtACS6        -ATTTTGTATCTCATGTAATTTTAACCGGGTGAATA--ATATGAATTT--------G-AA
Mic50867      ------------------------------------------------------------
CsACS         -TTTTTATGCTTCGTGCATTTGTAATTCTTTTTAAAATTATTGTACTG--------T-TC
Mic18584      ----TCGTAATTCCAATTTCAATAAATCTATGTGTACCTTGT-----------------T
TcACS10       TTCTGTATCATTGATGAAGAGAGAGAGAGGGACGGAGGGAGGGCAGGGATTTAAAGTTTT
Mic22459      ------------------------------------------------------------

Mic48715      ------------------------------------------------------------
Mic38692      ------------------------------------------------------------
Mic32921      ------------------------------------------------------------
AtACS6        ATCAGAATTTGTTT----------------------------------------------
Mic50867      ------------------------------------------------------------
CsACS         GTGTGTATTTTTTCG-----TTGGTGAAAAAGTCTATCATTTGC----------------
Mic18584      TTAAGTAT----------------------------------------------------
TcACS10       TCAAGTAAATTTTGAAGGAGACGGTCAAAAATACATGCCATTGTCACAAATTAAGGACCA
Mic22459      ------------------------------------------------------------

Mic48715      ---------------------------------
Mic38692      ---------------------------------
Mic32921      ---------------------------------
AtACS6        ---------------------------------
Mic50867      ---------------------------------
CsACS         ---------------------------------
Mic18584      ---------------------------------
TcACS10       AAACCTGAAGAACAGTAAAGTTGACAGACAGAC
Mic22459      ---------------------------------
```

Figure S2b: Multiple sequence alignment of different contigs representing 1-aminocyclopropane-1-carboxylic acid oxidase. Sequence with green background shows the start and stop codons. Mi stands for *Mangifera indica*, Cs stands for *Citrus sinensis*.

```
Mic33441      ------------------------------------------------------------
Mic18647      ------------------------------------------------------------
Mic23853      ------------------------------------------------------------
Mic23735      ------------------------------------------------------------
CsACO         TGGCTTGCTTTTTAGCCAAGTGGATGGTTGTGCTGGAGCCGTACTGGTG-CACATTGACA
Mic26899      ------------------------------------------------------------
Mic46112      ------------------------------------------------------------
Mic31832      ------------------------------------------------------------
Mic22984      ----------------------------------GTGTCCCTATAAATACCACCCCAACC
MiACO         ------------------------------------------------------------


Mic33441      ------------------------------------------------------------
Mic18647      --------------------------------------------TCTGACA-AAGTTA--
Mic23853      -----ATCTCTTTTA-AATTATTTGTTCACT------ACTTGACTTTGGAAGATAAAG--
Mic23735      ------------------------------------------------------------
CsACO         TCCATGTGCTTCTCA-ATGTTTGCCTTCACGCGTCGAAGTTGGATTTGAAAGAAAAAG--
Mic26899      --------------GTAAAG-------CAG----ACCAGTAAAGTGCGAAAGAAAGAA--
Mic46112      ----------------------AACTTCAAATAGATTACTTTCAAAGAAGAGAGAGAGAA
Mic31832      ------------------------------------------------------------
Mic22984      TCCACCCTCTTTGCATTAAGAAACACACAGCACAGCTTGTTGAGAGAAAGAGTCAGCCAT
MiACO         --------------------------CGGCACGAGCTTGTTGAGAGAAAGAGTCAGCCAT


Mic33441      ------------------------------------------------------------
Mic18647      ---TGAGTCGCCTTCACATCAATCATAACTTTG-----------------------TCTT
Mic23853      ---TGAAGTAACAA----ACAATGTCGG----------------------------TATA
Mic23735      ------------------------------------------------------------
CsACO         ---CAGATGAACGAAAAACCAATTATGACGTTTATTTACTTCTGGAAGGAAAATTATTTG
Mic26899      -------AAATGGAAGCTCTGAAACTCCCTATCATAGACCTCTCT---------TCAGCG
Mic46112      AGAAAGATGGAGAAGAACATGAAGTTTCCAGTAGTAGACTTGTCCAAGCTCAATGGGGAA
Mic31832      ------------------------------------------------------------
Mic22984      TTGTGAGAGATGGAGAGCTTGAGTTTCCCAGTCATTAACTTAGAGAAACTAAATGGTGAG
MiACO         TTGTGAGAGATGGAGAGCTTGAGTTTCCCAGTCATTAACTTAGAGAAACTAAATGGTGAG


Mic33441      ------------------------------------------------------------
Mic18647      ATCCCCTTTCTCGTTACAATCACTTCTCCCAACAGTTATCCTCAACACTCAGAATCGCAG
Mic23853      AATCTCAACG-ATTGTCCATAAACTTCA-CCATCATTATCTT---CAT-TCTCATCATTT
Mic23735      ------------------------------------------------------------
CsACO         AATATCTCTG-CTGGAAGTTAACATCCTTGAATCCTTGACTG---CGAGTGCAATGGTGT
Mic26899      GACCGCTTCTCCGCT---GCCCAATCAATCCGTCAG---------GCATGCGTAGAATAT
Mic46112      GAGAGAGACCAAACC---ATGGCTCTAATCAATGAA---------GCTTGTGAGAATTGG
Mic31832      ------------------------------------------------------------
Mic22984      GAGAGAGCTGCCACC---ATGGAGAAGATTAAAGAT---------GCTTGTGAGAACTGG
MiACO         GAGAGAGCTGCCACC---ATGGAGAAGATTAAAGAT---------GCTTGTGAGAACTGG


Mic33441      ------------------------------------------------------------
Mic18647      GTCCTCAATCAACACGAGCGAAAATGGTGACCACTATTCCACATGAAGCA-------CAG
Mic23853      TTCTTGTCTCAGTGAACCAGAATATGGACA---------------TTTCA-------AAT
Mic23735      --------------------------------------------AACCCA-------TCT
```

```
CsACO          GATCTCTATCA-AGCAATAAACAATGGATATCTCCGATGCCAGTAGAACG-------ACC
Mic26899       GGTTTCTTTTATCTGGTGAATCATGGAGTAGATGAGGAATTGCTTGGAACCATGTTTGAT
Mic46112       GGCTTCTTTGAGATAGTGAACCATGGATTACCACATGACTTAATGGACAAGATCGAGAAG
Mic31832       ------------------------------------------------------------
Mic22984       GGCTTCTTTGAGTTGGTGAACCATGGGATACCCATTGAGGAACTTGACACTCTGGAGAGG
MiACO          GGCTTCTTTGAGTTGGTGAACCATGGGATACCCATTGAGGAACTTGACACTCTGGAGAGG


Mic33441       ------------------------------------------------------------
Mic18647       CTGACGATGGAACCCAACCGCGATCGAACAAGTGAATTAAAAGCTTTCGAT-GACACAAA
Mic23853       GCCGTTGAAGCATCAGATTATGACAGAGCCGAAGAGCTTAAGAGCTTTGATGC-CACAAA
Mic23735       TCTGAAACTGTATCGGATTATGATCGAGCTAGAGAGCTTCAAGCTTTTGATGACAACAAA
CsACO          ACGCAAACTGTGACGGACTACGACAGAGCTAAAGAGGTTCAGGCTTTTGATGA-TACAAA
Mic26899       CAGAGTAGGAAATTCTTCTCACTTCCCTTGGCACACAAGATGACTTTGGCTCGCAAAGAA
Mic46112       ATGACAAAGGACCATTACAAGACATGCCAAGAA---------------------------
Mic31832       -----------------------------GGAA---------------------------
Mic22984       AAGACAAAAGAGCACTACAGAAAATGTATGGAA---------------------------
MiACO          AAGACAAAAGAGCACTACAGAAAATGCATGGAA---------------------------


Mic33441       ------------------------------------------------------------
Mic18647       AGCCGGCGTTAAAGGA-CTCGTTGATGCCGGAATTGACAAGATTCCCAGGATATTCTTTA
Mic23853       AGCTGGAGTTAAAGGA-TTAGTAGACTCTGGGATTGTGAAAATTCCCAGGATATTCATTC
Mic23735       AGCCAGCGTCAAAGGA-CTAGTAGATGCTGGAATTCTGAATGTTGCAAGGATATTCATTC
CsACO          AGCCGGTGTCAAAGGA-TTAGTGGATGCTGGAATTGTGAATATTCCGAGGATATTCATTC
Mic26899       CATAGAGGTTACACGCCTCT--CTATGCTGAGAATCTT--------GACCCC--------
Mic46112       --CAAAAGT---------------------------------------------------
Mic31832       --GAGAGATTCAAGGAATCGATTAAGAACAGAGGTCTT--------GACTCTCTTCGC--
Mic22984       --CAAAGGTTCAAGGAACTTGTGGCTAGTAAAGCTTTG--------GAAGGTGTTCAA--
MiACO          --CAAAGGTTCAAGGAACTTGTGGCTAGTAAAGCTTTG--------GAAGGTGTTCAA--


Mic33441       ------------------------------------------------------------
Mic18647       GCCCACCCTATGCTAATCAGGTTTCTAGTGCTACAGAATA---------TAAGTTTCCGA
Mic23853       GACCAGCGGATGAACTAGCCCAGGAGAAG---TTGACCAGTCATCAACACAACTTTCAAG
Mic23735       GCCCACCTTCTCCTAATGAGCTTGTTCACAGGTCAAATTATCACAAGAACAATCTGCAGG
CsACO          GACCGCCTGAGGAGCTTGTCGAGGAGTTG------ACCAGTCATCAGACCAATTCTCAAG
Mic26899       -----TCTTCTTCTTCTAAAGGTGACTCAAAAGA---------------GAGTTTCTACA
Mic46112       ------------------------------------------------------------
Mic31832       -----TCTGAAGTCAACGACGTTGACTGGGAATC---------------CACTTTCTACC
Mic22984       -----ACTGAGATCAAAGATATGGACTGGGAGAG---------------CACCTTCTATG
MiACO          -----ACTGAGATCAAAGATATGGACTGGGAGAG---------------CACCTTCTATG


Mic33441       ------------------------------------------------------------
Mic18647       TTATCGACCTGGGAAGACTTGATAAAGATCCTGGTCAACGCAAGGAGATTGTTGAAAAAG
Mic23853       TACCGGTCGTAGATCTTGATGGCATCAAAGACGACAGGCTCAAGGAGATTGTTGATGAGG
Mic23735       TCCCTCATA--------------TATCTTTGAGGCAAACACGAGGAGGTTGCTGATCAGG
CsACO          TACCGGTTATCGATCTTGATGGCGTCAGAGGCAATAAGCTTGAGGAGATTGTTGATCAAG
Mic26899       TTGGTCCTTTGGAAGGA------ACTCTTAGTAGCTTGAATCAGTGGCCCTCTGAAGAAG
Mic46112       ------------------------------------------------------------
Mic31832       TCAAGCACCTTCCCGTC------T------CTAATATCTCCGATGTCCCTGATCT---CG
Mic22984       TTCGCCATCTCCCTCAG------A------CAAACTTAAATGAACTCCCAGATCT---CG
MiACO          TTCGCCATCTCCCTCAG------A------CAAACTTAAAGTGCACCCCCGATCT---CG


Mic33441       ------------------------------------------------------------
Mic18647       TTCGAAATGCTTCGGAGACGTGGGGGTTCTTTCAGGTTATCAATCATGGGATTCCTGAGA
Mic23853       TCCGGGTTGCATCCGAGACATGGGGATTCTTTCAAGTTGTGAATCATGGGATCCCCTTAA
Mic23735       TTAGAAGTGCATCCGAGACATGGGGTTTTTTTCAAGTTGTGAATCATGGGATCACCTTGA
```

```
CsACO         TTCGGGCTGCAGCAGAGACATGGGGCTTCTTTCAAGTGGTGAATCACGGTATTCCCTTAA
Mic26899      TT-------------------TTGCCATCTTGGAGATCCACAATGGACCTTTTCTATAAA
Mic46112      ------------------------------------------------------------
Mic31832      AC----------------------GACGATTACAGAACGTTAATGAAAGACTTCGCCGGA
Mic22984      AT----------------------GATGAATACAGGAAGGTGATGAAGGAATTTGCATCA
MiACO         AT----------------------GATGAATACAGGAAGGTGATGAAGGAATTTGCATCA


Mic33441      ------------------------------------------------------------
Mic18647      GCGTTCTTGAGGAGATGAAGGATGGGGTTCGCAGGTTTTATGAGCAAGATTTTGAGGTGA
Mic23853      CTGTGTTAGAAGAAGTGCTTCATGGGGCACGAAAATTCCATGAACAAGACCTTGAGTTGA
Mic23735      ATGT--------------------------------------------------------
CsACO         ATGTGTTGGAAGAGATTATTGAAGGGATACGCAAGTTTAATGAACAAGACGTTGAGTTGA
Mic26899      AAAGTCCTG--GATATTGGATGTAGATTAATCCATTTAATAGCTCTAGCTTTGGAATTGG
Mic46112      ------------------------------------------------------------
Mic31832      AAGATAGAG--AAGTTGTCGGAGGAGCTACTGGATCTGCTGTGCGAGAATCTCGGTTTAG
Mic22984      AAATTGGAG--AAACTAGCAGAGGAACTGCTGGACTTGTTGTGTGAGAACCTTGGATTGG
MiACO         AAATTGGAG--AAACTAGCAGAGGAACTGCTGGACTTGTTATGTGAGAACCTTGGATTGG


Mic33441      ------------------------------------------------------------
Mic18647      AAAAACAATGGT---------------ACACCCGTGATTTTACCAGAAAGTTGATCTACA
Mic23853      AGAAAGAGTTAT---------------ATTCTCGTGATCCAAAGAGAGGCGTCGTATTCT
Mic23735      ------------------------------------------------------------
CsACO         AGAAAGAGTTTT---------------ATACCCGTGACCGGGCAAGAAATGTCAGATTCA
Mic26899      ATGAAGATTTCTTTGAGAAAGTGGGAGCATTGGATATGCCATCTGCATTTCTACGCTTGA
Mic46112      ------------------------------------------------------------
Mic31832      AGAAGGGTTATTTAAAAAAGGTGTTTTACGGGTCGAAAAGACCGACTTTTGGAACCAAAG
Mic22984      AGAAAGGTTACCTGAAGAAGACCTTCTATGGCTCAAAGGGGCCAACTTTTGGCACCAAGG
MiACO         AGAAAGGTTACCTGAAGAAGACCTTCTATGGTTCAAAGGGGCCAACTTTTGGCACCAAGG


Mic33441      ------------------------------------------------------------
Mic18647      -ATAGCAATTTTGACTTATACACAGGACCAGTGACGAACTGGCGGGATACCATGTCTTGT
Mic23853      -TCAGCAATCCAGATCTGTATCGATCTCCAACTGCTAATTGGAGGGATACTTTGGTTATT
Mic23735      ------------------------------------------------------------
CsACO         -ACAGCAACTTTGATCTGCATTACTCCCGGACAGCTAATTGGAGGGACACGTTGACAATT
Mic26899      TACACTATCCAGGCAAATTAGATTTGTCTGGTGAAGAAATATATGGTGCTTCTGCTCATT
Mic46112      ------------------------------------------------------------
Mic31832      -TCAGCAATTATCCACCTTGTCCTAATC--CGGACCTAGTCAAGGGTCTCCGAGCCCACA
Mic22984      -TTAGCAACTACCCACCATGTCCACAAC--CCGATTTGATCAAGGGCCTCCGAGCCCACA
MiACO         -TTAGCCACTACCCACCATGTCCACAAC--CCGATTTGATCAAGGGCCTCCGAGCCCACA


Mic33441      ------------------------------------------------------------
Mic18647      TA---CATGGCTCCTCATCCTCCCCAGTCAGAAAATTTACCAGCTGTTTGCAGAGATATA
Mic23853      TCTACTTTAGCTTCGAAGCAATTTGATCCAAGTAATATACCAGAAGTCTGCAGAGACAGT
Mic23735      ------------------------------------------------------------
CsACO         TCTGCTTTAGCTTCCACCGATTTAGACCCCAACGAATACCCAGAAGTTTGCAGAGATGCT
Mic26899      CAGATTATG-----GAATGATCACTCTTCTAGCTACTGATGGGATTCC------------
Mic46112      ------------------------------------------------------------
Mic31832      CCGACGCCGGCG--GCATC-----------------------------------------
Mic22984      CCGACGCCGGTG--GCATCATCTTACTCTTCCAGGATGATAAGGTGAG------------
MiACO         CCGACGCCGATG--GCATCATCTTACTCTTCCAGGATGATAAGGTGAG------------


Mic33441      ------------------------------------------------------------
Mic18647      CTAGTGGAGTACTCGAAGCAAGTAATGGAACTTGGCTATGTATTGTTCGAACTATTATCA
Mic23853      TTAATGGCGTATATAAGAACTATTTCAAAGCTGGGGAACACTCTATTTGAACTGTTATCG
Mic23735      ------------------------------------------------------------
```

```
CsACO        GTGAGAGAGTACATTAAAAATGTCACAAAATTGGGTGAAACTCTGTTTGAATTATTATCA
Mic26899     ------------------------------------------------------------
Mic46112     ------------------------------------------------------------
Mic31832     ------------------------------------------------------------
Mic22984     ------------------------------------------------------------
MiACO        ------------------------------------------------------------

Mic33441     ------------------------------------------------------------
Mic18647     GAGGCTCTTGGTTTGAAACCAAGTCACCTGAAAGACATGAATTGTGCTGAGGGGCTTGTA
Mic23853     ATGGCTTTAGCGCTGAAACCTGAACATTTAAAAGAGATGGATGCACTGAAGGATTTAAT
Mic23735     ------------------------------------------------------------
CsACO        CTGGCTCTTGGGCTGAAGGCTGAACATTTACAAGAGATTGGATGCCCTAAAGAATACACC
Mic26899     AGGACTACAGGTTTGTAGGGAGA-------------------------------------
Mic46112     ------------------------------------------------------------
Mic31832     ------------------------------------------------------------
Mic22984     CGGCCTCCAGCTT-CTG-------------------------------------------
MiACO        CGGCCTCCCAGCTTCTG-------------------------------------------

Mic33441     ------------------------------------------------------------
Mic18647     TTACTTTGCCATTACTATCCACCATGCCCACAGCCAGAATTAGCAATCGGCACATCCAGG
Mic23853     ATATCTTGCCTCTACTATCCATCGTGCCCCCAGCCAGATCTCACCTTGGGAGCTACCAAA
Mic23735     ------------------------------------------------------------
CsACO        CTTCCCTGCCACTACTATCCATCTTGCCCTCAGCCAGATTTGACATTGGGAGCTACCGGG
Mic26899     ------------------------------------------------------------
Mic46112     ------------------------------------------------------------
Mic31832     ------------------------------------------------------------
Mic22984     ------------------------------------------------------------
MiACO        ------------------------------------------------------------

Mic33441     ------------------------------------------------------------
Mic18647     CACTCGGACAATGACTTTCTCACTGTACTTCTACAAGACCATGTTGGTGGCCTCCAAGTT
Mic23853     CACACTGATCCTTCATTCTTTACTATAGTTTTGCAAGACCAAATCGGTGGTCTTCAAGTC
Mic23735     ------------------------------------------------------------
CsACO        CATTCAGACCCTTCATTTCTTACCATTCTTCTGCAAGACCAAATCGGCGGCCTTCAAGTC
Mic26899     -------------------------------------------------------AATTC
Mic46112     ------------------------------------------------------------
Mic31832     ------------------------------------------------------------
Mic22984     ------------------------------------------------------------
MiACO        ------------------------------------------------------------

Mic33441     ----------------------------------------AAGCACTTATCTTTAACGTA
Mic18647     CTTCATCAAAACCAGTGGGTTGATGTGCCTCCCACCCCTGGTGCTCTTGTGGTTAACATT
Mic23853     TTCCATGAAAATCAGTGGGTTAATGTTGAACCCATTGCTGGAAGCTTGGTGGTAAATATA
Mic23735     ------------------------------------------------------------
CsACO        TTTCATGACAATCAATGGGTTGGTGTCCAACCCATTGTGGGAGGCTTAGTGGTAAACATA
Mic26899     AAAGAATCAAGGGCATGGGAGGAGGTGCCAAGTATCAAGGGGGCATTCATTGTTAATATT
Mic46112     ------------------------------------------------------------
Mic31832     ------------------------------------------------------------
Mic22984     -AAAGATGGGC--AGTGGATTGATGTGCCCCCTATGCGCCACTCCATTGTGGTTAACCTT
MiACO        -AAAGATGGGCAGTGGATATTGATGTGCCCCCAATGCGCCACTCCATTGTGGTTAACCTT

Mic33441     GGAGATTTCCTCCAGCTTATCTCGAATGACAAGTTTGTAAGCATGGAGCATAGAATTTTG
Mic18647     GGAGATCTCATGCAGCTAGTATCAAATGACAAATTCATAAGCGTGGAGCATAGAGTACTG
Mic23853     GGGGACTTTCTCCAGGTTATATCGAACAACAAGTTTAAAAGTGTTGACCACAGAGTCCTC
Mic23735     ------------------------------------------------------------
```

CsACO	GGGGACTTCCTCGAGGTTGTATCAAACGACAAGTTCAAGAGTGTTAACCACAGAGTAGTC
Mic26899	GGGGACATGATGGAGAGGTGGACTAATTGTTTATTTCGGTCAACCCTTCATAGAGTGATG
Mic46112	--
Mic31832	--
Mic22984	GGTGACCAGCTTGAGGTTATCACCAATGGCAAATACAAGAGTGTGGAGCACCGAGTAGTT
MiACO	GGTGACCAGCTTGAGGTTATCACCAATGGCAAATACAAGAGTGTGGAGCACCGAGTAGTT

Mic33441	GCAAATGGAGGTGAAGAGCCGCGCATTTC-------------------------------
Mic18647	GCGAACCGGGTAGGGCCAAGAATATCAGT-------------------------------
Mic23853	GCCAGTGGATTTGTTCCAAGGATTTCTGT-------------------------------
Mic23735	--
CsACO	GCCGGTCATGTCGGTCCAAGGGTTTCCGT-------------------------------
Mic26899	CCAACAGGACAAGAACGCTACTCTGCTGCATTCTTTTTGGATCCCAACAGTAACTGTGTA
Mic46112	--
Mic31832	--
Mic22984	GCTCAAAGAGATGG-------------------CAACGGCCGCATGTCAATAGCTTCATT
MiACO	GCTCAAAGAGATGG-------------------CAACGGCCGCATGTCAATAGCTTCATT

Mic33441	--
Mic18647	--
Mic23853	--
Mic23735	--
CsACO	--
Mic26899	GTGGAATGCCTGGAAAGTTGTTGCAGTGAATCCTGTCCCCCAAGATTTCCCCCCATCCGC
Mic46112	--
Mic31832	--
Mic22984	CTACAACCCTGGCAGTGATGCAGTTATCTATCCAGCACCACCATTGTTGGAAAAAGAAGC
MiACO	CTACAACCCTGGCAGTGATGCAGTTATCTATCCAGCACCACCATTGTTGGAACCAGAAGC

Mic33441	-----GGT----------CGCTTGTTTCTTTGTGCATACTTTTACTTCACCAAGTTCGAG
Mic18647	-----GGC----------GAGCTTTTTCAGCACAGATCTTCAGTCAGATTGCTCATCGAC
Mic23853	-----AGC----------ATGCTTTTTTACTGGACATGCTACTGG------AACCCCAAA
Mic23735	--
CsACO	-----GGC----------ATGCTTTTTCATGGGACATAATGCAGA------AATCCCAAA
Mic26899	AGTGGGGACTACTTTAAAGAGCGGTTAAGACTTACATATGGATCA**TAA**GAGGAGCTTTCA
Mic46112	--
Mic31832	--
Mic22984	AGAGGAGAAGAA--ACAAGTGTACCCAAAATTTGTTTTTGAAGACTACATGAAGCTGTAT
MiACO	AGAGGAGAAGAA--ACAAGTGTACCCCAAATTTGTTTGTGAAGACTACATGAAGCTGTAT

Mic33441	AGTATATGGACCCATTAAAGAGCTTCTGTCTGAGCTAAACCCTCCA--------------
Mic18647	AGTTTATGGACCAATCCAAGAGTTGTTATCAGAAGAGAATCCTCCA--------------
Mic23853	GCTCTATGGACCAATTAAAGAGTTAACATCAGATGAAAATCCACCT--------------
Mic23735	--
CsACO	GTCTTATGGGCCGATCAAAGAGTTGACATCTGAAGAAAATCCTCCT--------------
Mic26899	GATTGGTGGTCGTATGCAAAAGTTAAAGTTTAAATACTTTCCCTTTTATAATGGGTGTCA
Mic46112	--
Mic31832	--
Mic22984	GCCGGCTTGAA----------GTTCCAGGCAAAAGAACC--CAGGTTTGAAGCCATGAAA
MiACO	GCCCGCTTGAA----------GTTCCAGGCAAAAGAACC--CAGGTTTGAAGCCATGAAA

Mic33441	---------------AAATACAGAGACACCACCTCGGAATCCTCCA---ATCACTATGTG
Mic18647	---------------AAGTACAGGGAAACCACAGTGAAAGACTATGTCGCATACTTTAAG
Mic23853	---------------GTTTACAGGGAATTTCTTGTTTCTGAATATTTCAACTTATATTTT
Mic23735	--

```
CsACO          ---------------ATTTACAGAGATTTTCTTCCAAGTGAATGTTTTAGCAAACGATTT
Mic26899       CCATTACAGCCCATATATAAATTTTAACCCTCAACATGTGCTTTT----CTGGGGATAAC
Mic46112       ------------------------------------------------------------
Mic31832       ------------------------------------------------------------
Mic22984       GCTGTTGAAACCACTGTTAATCTGGGCCCAATTGCCACTGCTT-A----ATTAAGAAAAG
MiACO          GCTGTTGAAACCACTGTTAATCTGGGCCCAATTGCCACTGCTT-A----ATTAGGAA---

Mic33441       GCTAGAAAACCTAATGGGAATTCTTCGTTGGACCATTTAA---------GGATCTGAAAC
Mic18647       GGAAAGGGTCTCGATGGGACTTCTGCGCTGCTTCATTTCA---------GGCTATAAGCT
Mic23853       GCTAAGGTAGTTGGTGATAAGTCTGTTCTTTGTCAATTCA---------AGCTGTGAAGA
Mic23735       ------------------------------------------------------------
CsACO          TCTATGGCGGTTGATGACAATTCTCTCCTCCATCAGCTCA---------AGCTATGAAGC
Mic26899       TGTATTTACCCTCCTGTTTTTATTCTATTAGTTCATATTCTGCGGTTTGAAATATGAAAT
Mic46112       ------------------------------------------------------------
Mic31832       ------------------------------------------------------------
Mic22984       AAAATGTAATTTACTGCTTAATTTTTGTGTGTTGATGTCGTGTG-----AAG-------C
MiACO          --AATGTAATTTACTGCTTAATTTTTGTGTGTTGGTGTGGTGTG-----AAG-------C

Mic33441       TTGAACCTATATCTCAGAGGT---------------------------------------
Mic18647       TCGCAAAGAAGATGAAATGAA----TATCTGGAT--------------------------
Mic23853       GGGAGAATACTTGAA--TAAA----GTTTGTAAATCTTGTACGAATTATTAATAGGATCA
Mic23735       ------------------------------------------------------------
CsACO          CGACAGTATTTGTAT--TAAT----TATTCGTGT-TTG----------------------
Mic26899       TTGGGCTTTGGTACAACAATATCA-TTCAATCAGGTGTGTTCAAATTCTTACTGGGTAGC
Mic46112       ------------------------------------------------------------
Mic31832       ------------------------------------------------------------
Mic22984       T------TGGTTATGAAGAAGTCAAAGCTTACTGCAGTATCCATATCATTACTATTAATA
MiACO          T------TGGTTATGAAGAAGTCAAAGCTTACTGCAGTATCCATAGTATTACTATTAATA

Mic33441       ------------------------------------------------------------
Mic18647       -AATTAAATAAATGGTTTTTCTGAAGT-AAAACAATTTAGCGCTTGTTTGGAATTGTTTA
Mic23853       TATTTGAATAAAGTTTGCAACTTTAATACAAACAATTAATATATTGAGGACTGCT----A
Mic23735       ------------------------------------------------------------
CsACO          -------------------------TTACCAACAATTTACAAATTGCGGAGCCCT----G
Mic26899       GTTT-AGTCACCG-------CCCAC--GGC--CAAGGTTTGGACTTGTGACTAATGAATC
Mic46112       ------------------------------------------------------------
Mic31832       ------------------------------------------------------------
Mic22984       ATTT-GTAGACAG-------CAACAGATGTAACATTATTTAATAATAATACTAATAGACC
MiACO          ATTT-GTAGACAG-------CAACAGATGTAACATTATTTTATAATAATACTAATAGACC

Mic33441       ------------------------------------------------------------
Mic18647       ATGTCTTGTCTATTTACATATATCCTACTTGTAACCATGTTCAAAACTATATA--TGATT
Mic23853       TTCTCAAGTGTTTCCAAATAAAGCTAA----A-----TATTTCAACAATTTGAGTCCTTA
Mic23735       ------------------------------------------------------------
CsACO          TTGTGAACTCTTTCCCAGTAATTCCGA----AAAAAATGTTTCAACTAATTAATAACGTA
Mic26899       GTGTCTAAAGTGAACACGTTAACCACA----C----GGCTCTAAAGTGAGT----CGCAG
Mic46112       ------------------------------------------------------------
Mic31832       ------------------------------------------------------------
Mic22984       TTGTCTCCCCTTCATTATTTTATATTT----T----AGATCTC-----------------
MiACO          TTGTCTCCCCTTCATTATTTTAAAAAA----A----AAAAAAA-----------------

Mic33441       ------------------------------------------------------------
Mic18647       TGTAGTTCTTGATGGGTGTGTTTTGTTGAAGGA-----------------ATTATTTCTT
Mic23853       ATTTCTTTCTGCAGTAGCCCTTCTGTTAACAGGAGTACTGGGTACCCTTGATCATGGTTC
Mic23735       ------------------------------------------------------------
```

```
CsACO        TGAATCGTCCAATGTTGCTCTTTACTT---------------------------------
Mic26899     AGGAAT-----GGGTGTCCCTTACGT-GTAAGACAACAACAGTACAGCGCGTTAATAAAG
Mic46112     ------------------------------------------------------------
Mic31832     ------------------------------------------------------------
Mic22984     AGAA--------ACTGTTCAT------AGAAGCAACA--------AAAATGATATAAAAT
MiACO        AAAA--------------------------------------------------------


Mic33441     -----------------------------------
Mic18647     ATTTCAATGTGACAAAATAA---------------
Mic23853     TTCTTGATGCTTCCAAATACAAATTAAGATTTG--
Mic23735     -----------------------------------
CsACO        -----------------------------------
Mic26899     ACGTCCTTTATAGCAACTCTACTTAAATCTCAACC
Mic46112     -----------------------------------
Mic31832     -----------------------------------
Mic22984     ATATCCTGGAGGGGAAATCTTG-------------
MiACO        -----------------------------------
```

Figure S3: Expression profile of contigs annotated as CTR1-like gene, as obtained from "Dashehari" transcriptome. Illumina sequencing was performed in triplicates, data is represented as mean with ±SE. P=0.01 for all data sets.

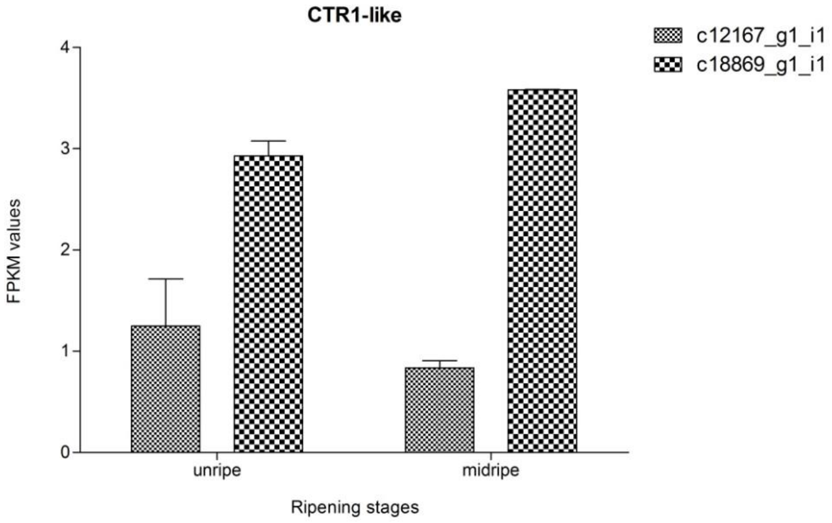

Figure S4: Expression profile of 14-3-3 proteins as obtained from "Dashehari" transcriptome. Illumina sequencing was performed in triplicates, data is represented as mean with ±SE. P=0.01 for all data sets.

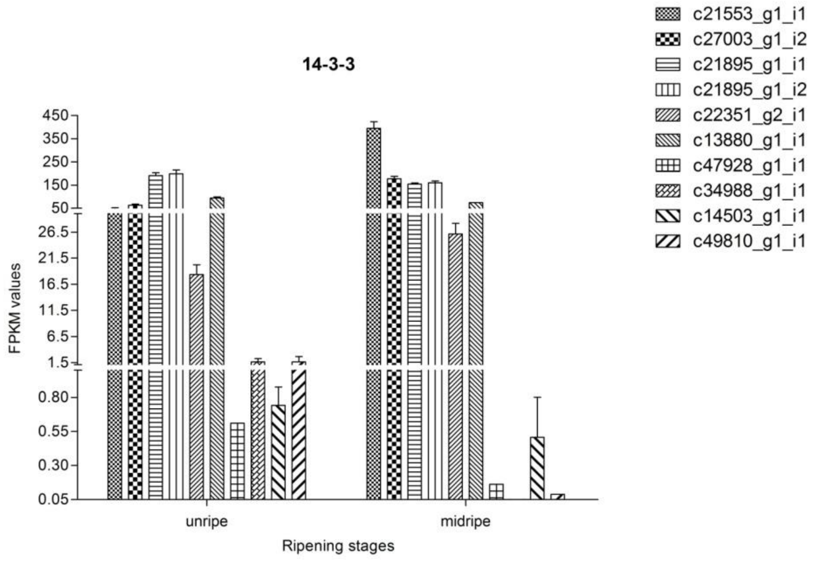

Figure S5: Expression profile of XTHs as obtained from "Dashehari" transcriptome. Illumina sequencing was performed in triplicates, data is represented as mean with ±SE. P=0.01 for all data sets.

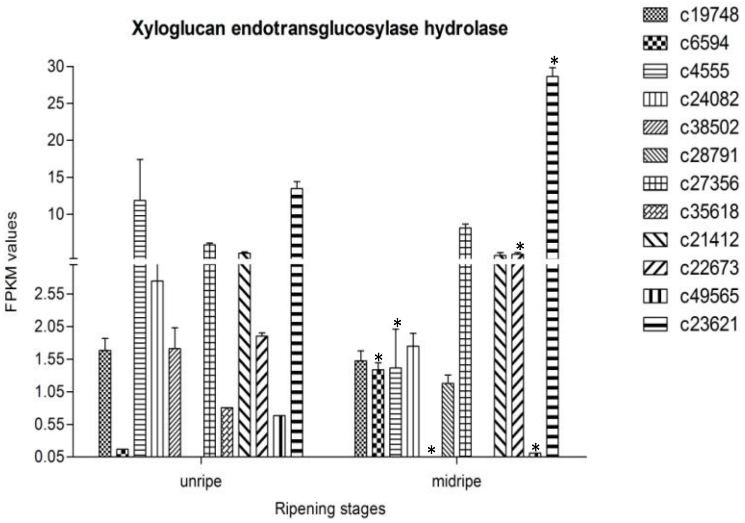

Figure S6: Expression profile of Galactosidases as obtained from "Dashehari" transcriptome. Illumina sequencing was performed in triplicates, data is represented as mean with ±SE. P=0.01 for all data sets.

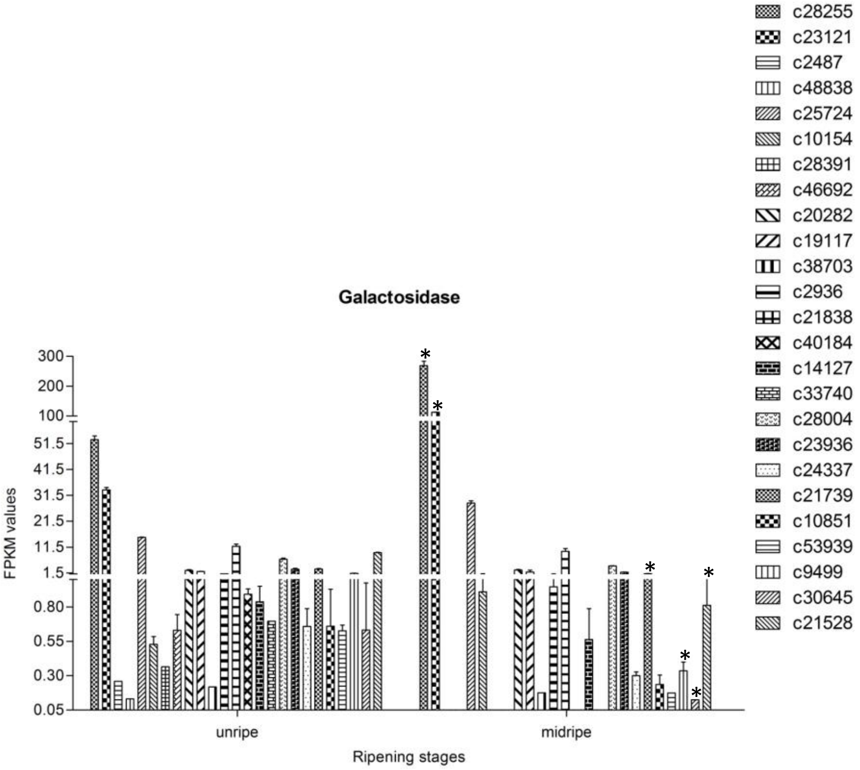

Supplementary Table S1: List of transcription factors differentially regulated in "Dashehari" Transcriptome. Statistical test method: hypergeometric test / Fisher's exact test, FDR correction method: Benjamini and Hochberg

Transcription Factor	Contig Id	Fold Change	P-Value	Transcription Factor	Contig Id	Fold Change	P-Value	Transcription Factor	Contig Id	Fold Change	P-Value
AP2	c20654	4.59	9.14E-06	CO-like	c20702	-2.11	1.40E-05	MYB	c13400	6.27	1.24E-09
AP2	c20549	4.41	9.62E-10	CO-like	c19931	-2.60	0.000109	MYB	c19378	5.33	4.21E-16
AP2	c20774	3.99	1.15E-07	CO-like	c46997	-4.05	0.001174	MYB	c12893	4.14	0.000108
AP2	c26537	-2.68	1.02E-05					MYB	c20105	4.11	1.80E-12
AP2	c21356	-2.77	6.59E-08	CPP	c26259	2.73	3.01E-07	MYB	c28201	3.74	1.09E-14
				CPP	c25544	-2.12	4.40E-05	MYB	c28275	3.69	9.83E-12
ARF	c26627	6.01	1.10E-07					MYB	c10187	3.61	1.43E-05
ARF	c16595	2.51	0.000149	DBB	c17666	3.77	1.32E-11	MYB	c24538	3.61	1.31E-07
ARF	c27449	2.46	1.43E-07	DBB	c21156	2.90	0.0007	MYB	c20754	3.52	5.59E-13
ARF	c8037	-2.13	0.007795					MYB	c19933	3.22	8.61E-05
ARF	c14938	-2.39	0.001625	Dof	c15088	6.95	7.94E-18	MYB	c19927	3.12	7.10E-10
ARF	c24111	-2.43	1.83E-06	Dof	c26661	2.23	5.12E-06	MYB	c20663	2.83	0.002193
ARF	c17688	-2.77	7.88E-09	Dof	c28339	-2.02	0.000222	MYB	c23810	2.82	1.69E-06
ARF	c18707	-3.01	1.83E-08	Dof	c20689	-2.17	7.51E-06	MYB	c10699	2.61	0.002324
ARF	c40252	-3.44	0.000449	Dof	c18525	-2.44	0.003018	MYB	c26445	2.40	3.57E-06
ARF	c14053	-3.49	1.31E-08					MYB	c16305	2.39	5.34E-05
ARF	c47020	-4.78	5.64E-06	E2F/DP	c25808	4.34	3.78E-10	MYB	c14521	2.31	1.58E-06
ARF	c29264	-5.34	2.89E-06	E2F/DP	c21553	2.99	3.23E-10	MYB	c26971	2.27	0.003938
				E2F/DP	c26154	2.33	0.000511	MYB	c16195	2.18	8.63E-05
B3	c27331	3.08	1.41E-09	E2F/DP	c17040	2.06	0.00021	MYB	c24559	2.17	3.25E-06
B3	c14230	3.07	0.000148	E2F/DP	c28256	-2.51	0.003558	MYB	c19825	-2.00	0.000179
B3	c20648	2.93	1.84E-08	E2F/DP	c24725	-3.28	0.00369	MYB	c15494	-2.02	0.004778
B3	c27457	2.43	2.19E-07	E2F/DP	c11407	-3.78	0.000475	MYB	c23064	-2.10	2.77E-05
B3	c22208	-2.04	2.30E-05					MYB	c22224	-2.23	2.05E-05
B3	c22402	-2.30	0.003436	ERF	c18034	4.44	1.80E-06	MYB	c21749	-2.47	6.40E-05
B3	c27650	-2.34	3.78E-05	ERF	c22322	3.40	1.80E-11	MYB	c20940	-2.49	1.31E-07
B3	c22349	-2.44	4.90E-06	ERF	c14742	2.54	0.005898	MYB	c17533	-2.50	1.16E-06
B3	c26456	-2.56	2.55E-05	ERF	c13537	2.34	0.008966	MYB	c6517	-2.63	0.002346
B3	c26537	-2.68	1.02E-05	ERF	c39355	2.24	0.000154	MYB	c31014	-2.68	0.007747
B3	c22693	-2.72	8.08E-05	ERF	c26290	2.16	1.70E-05	MYB	c24893	-2.73	0.004014
B3	c911	-2.73	2.25E-08	ERF	c16914	-2.01	0.000416	MYB	c17846	-2.86	0.007786
B3	c19506	-2.78	2.48E-07	ERF	c20423	-2.18	0.000616	MYB	c16899	-2.86	2.04E-08
B3	c15180	-2.99	0.000704	ERF	c21896	-2.19	3.47E-05	MYB	c22468	-2.92	2.36E-07
B3	c16940	-3.24	8.55E-08	ERF	c3792	-2.25	0.000421	MYB	c17816	-2.96	9.01E-10
B3	c18003	-3.56	3.54E-08	ERF	c24624	-2.32	1.22E-05	MYB	c11814	-3.03	0.005645
B3	c29400	-4.30	0.002278	ERF	c17402	-2.34	0.000637	MYB	c29815	-3.09	0.00988
				ERF	c26938	-2.37	5.75E-07	MYB	c47661	-3.66	4.17E-07
BBR-BPC	c27154	2.17	0.004457	ERF	c22035	-2.42	8.30E-05	MYB	c12114	-3.82	0.000119
BBR-BPC	c22046	-2.08	0.000415	ERF	c16154	-2.50	0.000209	MYB	c49037	-4.02	0.008335

				ERF	c39593	-3.00	0.000126	MYB	c14902	-4.16	0.000334
BES1	c26524	4.19	1.93E-17	ERF	c32799	-3.38	0.005117	MYB	c40482	-4.33	0.00284
BES1	c27105	3.99	5.29E-14	ERF	c47790	-3.79	1.79E-07	MYB	c29858	-4.54	3.98E-06
BES1	c19792	2.49	3.96E-07	ERF	c15711	-3.83	0.000467	MYB	c5847	-4.74	7.67E-11
BES1	c29669	-5.12	2.28E-05	ERF	c29357	-3.88	2.79E-07				
				ERF	c40854	-4.37	0.00023	NAC	c27045	5.33	7.68E-19
bHLH	c13381	7.23	1.43E-13	ERF	c89	-4.40	0.001618	NAC	c22341	2.98	2.68E-06
bHLH	c26412	6.00	2.94E-12					NAC	c20364	2.67	0.003985
bHLH	c14886	4.32	3.70E-14	FAR1	c17956	3.13	0.000237	NAC	c24250	2.33	0.001341
bHLH	c23922	4.18	2.54E-15	FAR1	c27689	2.43	1.21E-06	NAC	c19395	2.04	0.000222
bHLH	c22822	3.34	3.07E-08	FAR1	c26453	2.30	0.001118	NAC	c27697	-2.04	0.000101
bHLH	c25955	3.11	1.04E-07	FAR1	c25919	2.22	1.68E-05	NAC	c521	-2.23	0.005115
bHLH	c28218	3.07	1.04E-07	FAR1	c23749	-2.02	0.001269	NAC	c35124	-2.33	0.003358
bHLH	c25901	3.05	1.29E-10	FAR1	c23329	-2.52	9.31E-05	NAC	c19974	-2.37	5.89E-06
bHLH	c22992	3.00	2.32E-07	FAR1	c20196	-3.63	8.48E-12	NAC	c25356	-2.45	0.003021
bHLH	c26099	2.85	3.17E-07	FAR1	c15178	-3.86	8.46E-06	NAC	c16898	-2.48	0.00643
bHLH	c26695	2.61	1.94E-06	FAR1	c19341	-4.33	6.23E-17	NAC	c13037	-2.62	0.000954
bHLH	c26084	2.60	0.002489	FAR1	c12712	-4.45	1.74E-09	NAC	c19293	-2.83	9.17E-06
bHLH	c16210	2.56	7.57E-07	FAR1	c7573	-5.43	4.40E-08	NAC	c25333	-2.93	1.25E-06
bHLH	c26383	2.34	0.005863					NAC	c30275	-3.13	0.000298
bHLH	c17722	2.27	9.73E-07	G2-like	c27805	4.14	1.85E-16	NAC	c4064	-3.14	0.000817
bHLH	c26766	2.25	0.000131	G2-like	c23959	2.37	1.67E-06	NAC	c19370	-3.21	3.66E-05
bHLH	c24970	2.21	2.03E-05	G2-like	c23223	-2.03	0.000772	NAC	c7377	-3.32	0.000726
bHLH	c20917	2.03	0.000168	G2-like	c23771	-2.11	0.009584	NAC	c45344	-4.21	0.002099
bHLH	c25979	-2.05	0.000155	G2-like	c25640	-2.39	7.13E-05	NAC	c40356	-4.23	8.01E-05
bHLH	c17021	-2.14	1.02E-05	G2-like	c25035	-2.68	6.68E-08	NAC	c47605	-4.79	0.000126
bHLH	c16739	-2.17	2.74E-05	G2-like	c20531	-2.69	1.43E-08	NAC	c2209	-5.76	6.75E-05
bHLH	c19647	-2.25	7.96E-06	G2-like	c14782	-3.16	1.17E-09				
bHLH	c17525	-2.38	0.000677	G2-like	c22084	-3.42	7.26E-11	NF-YA	c15427	4.03	7.90E-07
bHLH	c48802	-2.44	0.002172	G2-like	c11284	-4.00	0.008421	NF-YB	c24426	-2.45	1.07E-05
bHLH	c15810	-2.51	0.00146	G2-like	c5885	-4.64	1.19E-08	NF-YB	c27034	-2.63	0.000119
bHLH	c30995	-2.56	0.002951	G2-like	c19857	-5.31	1.48E-14	NF-YB	c27566	-3.07	2.22E-10
bHLH	c15050	-2.56	1.18E-05								
bHLH	c24379	-2.77	2.17E-05	GATA	c24160	3.16	1.42E-10	Nin-like	c17891	-2.54	4.11E-06
bHLH	c31267	-2.86	0.000286	GATA	c24595	3.03	8.65E-09	Nin-like	c9823	-3.54	0.000128
bHLH	c39894	-2.87	0.000283	GATA	c21351	2.22	4.58E-06				
bHLH	c13442	-3.07	0.001808	GATA	c15195	-3.10	8.77E-05	S1Fa-like	c15124	3.04	0.000236
bHLH	c13182	-3.14	0.000766	GATA	c48183	-3.91	0.000427	S1Fa-like	c17182	2.31	0.00417
bHLH	c23082	-3.16	4.90E-09					S1Fa-like	c17488	-2.16	8.86E-05
bHLH	c20604	-3.25	0.007869	GeBP	c22960	2.54	6.92E-07	S1Fa-like	c18033	-2.16	0.001128
bHLH	c17726	-3.28	0.000808	GeBP	c17211	2.02	3.76E-05	S1Fa-like	c16436	-2.22	0.003601
bHLH	c13973	-3.30	2.81E-05	GeBP	c12761	-2.32	0.002023	S1Fa-like	c12808	-2.37	0.001783
bHLH	c7801	-3.43	0.002814	GeBP	c20169	-2.68	0.002979	S1Fa-like	c12831	-3.23	0.001284
bHLH	c23522	-3.50	6.90E-06	GeBP	c48774	-3.12	0.000978	S1Fa-like	c19511	-4.18	7.92E-11
bHLH	c11372	-3.52	0.001108	GeBP	c14779	-3.88	0.008991				
bHLH	c9639	-4.12	4.50E-08					SBP	c28298	3.54	0.004735

bHLH	c39202	-4.38	0.002178	GRAS	c18816	2.59	1.48E-05	SBP	c16882	2.81	4.11E-06
bHLH	c12911	-4.57	4.31E-08	GRAS	c26439	-2.15	0.009287	SBP	c26136	2.14	2.36E-05
				GRAS	c3690	-2.31	0.003408	SBP	c23631	-2.06	0.000112
bZIP	c19260	5.13	1.28E-12	GRAS	c18601	-2.39	0.007934	SBP	c21236	-2.08	0.008088
bZIP	c28930	4.70	0.001029	GRAS	c47477	-4.11	1.28E-05	SBP	c14207	-2.78	5.48E-05
bZIP	c16021	3.99	0.00067					SBP	c19391	-3.23	2.97E-06
bZIP	c26902	3.64	1.22E-13	GRF	c9750	-3.21	6.18E-05	SBP	c19199	-3.28	1.89E-06
bZIP	c12658	2.39	0.007156					SBP	c25157	-3.29	1.24E-11
bZIP	c18448	2.30	0.005522	HB-other	c23711	6.08	1.95E-19	SBP	c37921	-3.69	0.00038
bZIP	c16810	2.26	0.00098					SBP	c21031	-4.01	0.008855
bZIP	c18188	2.22	2.35E-05	HD-ZIP	c27147	7.80	4.75E-39	SBP	c6332	-4.34	1.84E-05
bZIP	c47027	-2.35	0.002336	HD-ZIP	c21799	2.67	1.58E-07				
bZIP	c38886	-2.43	0.002194	HD-ZIP	c15099	2.22	0.000302	SRS	c27836	-2.67	2.65E-08
bZIP	c27674	-2.88	1.94E-09	HD-ZIP	c242	2.09	0.000733	SRS	c38539	-3.96	6.20E-07
bZIP	c21681	-3.00	0.000996	HD-ZIP	c18610	-2.15	0.001787	SRS	c15078	-4.13	9.43E-05
bZIP	c22945	-3.04	2.11E-08	HD-ZIP	c21215	-3.42	0.000814	SRS	c25337	-4.41	2.98E-14
bZIP	c4943	-3.33	0.000283	HD-ZIP	c18876	-4.06	8.10E-07	SRS	c12997	-4.69	0.000442
bZIP	c25943	-3.82	1.57E-13	HD-ZIP	c36147	-5.23	0.001444				
bZIP	c16361	-3.91	0.002139					TALE	c22649	-2.35	7.51E-07
bZIP	c3708	-3.97	0.000626	HSF	c22315	6.04	7.16E-24				
bZIP	c3742	-4.72	1.32E-08	HSF	c19819	5.32	2.52E-12	TCP	c16404	3.37	4.82E-06
bZIP	c37802	-4.81	0.006561	HSF	c13753	4.42	2.66E-08	TCP	c25966	2.67	6.15E-07
bZIP	c17489	-4.91	1.09E-11	HSF	c22946	3.54	7.86E-05	TCP	c17077	2.28	9.48E-06
				HSF	c25935	2.67	1.68E-08	TCP	c18280	-2.06	0.00263
C2H2	c1743	4.89	0.000297	HSF	c12716	2.66	7.71E-05	TCP	c49619	-2.36	0.004143
C2H2	c26690	4.45	0.003527	HSF	c48447	2.56	0.000348	TCP	c20629	-2.85	0.007794
C2H2	c26324	4.20	4.97E-12	HSF	c28136	2.52	0.000955	TCP	c14129	-3.37	4.59E-08
C2H2	c26506	4.19	1.57E-08	HSF	c12474	2.39	0.007883	TCP	c17023	-3.40	1.03E-05
C2H2	c24402	2.94	3.97E-06	HSF	c15168	2.16	6.23E-06	TCP	c15361	-3.56	4.87E-05
C2H2	c7129	2.76	0.000348	HSF	c23401	2.10	5.83E-06	TCP	c21230	-4.05	0.005961
C2H2	c22052	2.60	5.75E-08	HSF	c27347	-2.10	8.75E-06	TCP	c12727	-4.84	0.00885
C2H2	c9678	-2.11	0.007163	HSF	c14611	-2.37	3.25E-06				
C2H2	c16603	-2.48	4.72E-05	HSF	c20763	-2.41	0.000172	Trihelix	c25016	2.07	5.49E-05
C2H2	c23304	-2.68	0.000787	HSF	c23628	-2.50	0.006359	Trihelix	c18144	-2.66	4.58E-07
C2H2	c18254	-3.06	5.80E-08	HSF	c38784	-2.54	0.007945	Trihelix	c9983	-3.16	0.000318
C2H2	c14072	-3.20	1.45E-06	HSF	c20594	-2.63	1.27E-05				
C2H2	c14527	-3.30	0.000176	HSF	c11148	-2.80	2.30E-07	VOZ	c27207	2.23	2.23E-06
C2H2	c16052	-3.70	3.23E-08	HSF	c11260	-2.84	1.17E-05				
C2H2	c49298	-3.97	8.87E-05	HSF	c22092	-3.71	7.12E-14	WRKY	c19116	4.27	7.73E-05
C2H2	c15820	-4.45	0.000966	HSF	c17282	-3.89	9.33E-10	WRKY	c24262	4.03	5.52E-07
				HSF	c9865	-4.51	5.23E-08	WRKY	c24607	3.86	1.05E-12
C3H	c27394	6.27	3.57E-09	HSF	c23695	-5.00	2.00E-16	WRKY	c21459	3.55	0.004002
C3H	c26607	5.99	7.72E-12	HSF	c9739	-5.33	1.47E-07	WRKY	c24824	3.26	4.64E-11
C3H	c27115	5.99	3.37E-08					WRKY	c29033	2.13	0.00928
C3H	c38016	5.58	7.63E-14	LBD	c22818	3.94	2.19E-09	WRKY	c19104	2.05	0.000295
C3H	c20086	4.63	4.80E-09	LBD	c12652	3.50	8.19E-13	WRKY	c15826	-2.16	0.000525

C3H	c19011	4.36	1.77E-06	LBD	c25564	2.92	3.47E-05	WRKY	c24766	-2.20	9.86E-05
C3H	c17885	3.20	4.93E-08	LBD	c20944	2.79	6.12E-06	WRKY	c14499	-2.56	5.98E-06
C3H	c20588	2.84	0.001595	LBD	c18705	2.03	1.73E-05	WRKY	c11420	-2.82	0.001501
C3H	c24932	2.67	5.82E-06	LBD	c18893	-2.70	0.000152	WRKY	c39468	-2.91	2.81E-05
C3H	c28054	-2.10	1.04E-05	LBD	c7897	-2.94	0.000163	WRKY	c15910	-2.98	1.39E-09
C3H	c5074	-3.54	0.008088	LBD	c15466	-3.92	6.68E-06	WRKY	c37886	-3.49	9.29E-05
C3H	c29409	-3.57	4.27E-07					WRKY	c9120	-3.57	0.000836
C3H	c20385	-3.77	1.30E-08	MIKC	c24309	-2.00	0.001044	WRKY	c7032	-3.67	7.53E-08
C3H	c25387	-3.87	4.56E-14	MIKC	c17204	-2.89	3.58E-09	WRKY	c12489	-3.85	0.004003
C3H	c49580	-5.01	5.39E-05	MIKC	c7819	-5.34	0.000987	WRKY	c4638	-3.97	8.01E-05
								WRKY	c40182	-4.19	0.002131
CAMTA	c39879	-2.75	0.000518	M-type	c14662	3.98	0.009274	WRKY	c14531	-4.68	4.54E-07
CAMTA	c37861	-3.72	7.35E-06	M-type	c27307	3.48	7.40E-13	WRKY	c7649	-4.80	3.07E-07
CAMTA	c47090	-4.24	0.003132	M-type	c14628	-2.68	0.0006	WRKY	c44035	-5.41	0.000511
CAMTA	c7320	-4.37	3.24E-07	M-type	c22970	-3.12	2.33E-10	WRKY	c14607	-6.63	6.12E-30
				M-type	c18938	-4.02	7.94E-13				
				M-type	c29112	-5.13	5.09E-09	ZF-HD	c25550	-1.83	0.00026
								ZF-HD	c3851	-3.67	0.009217

Supplymentary table S2: Pathway enrichment against Arabidopsis

#Term	Database	ID	Input number	Background number	P-Value 0.1
glutathione-mediated detoxification II	BioCyc	PWY-6842	5	70	0.000192
S-adenosyl-L-methionine cycle II	BioCyc	PWY-5041	3	23	0.00082
jasmonic acid biosynthesis	BioCyc	PWY-735	3	33	0.00214
Phenylpropanoid biosynthesis	KEGG PATHWAY	ath00940	7	201	0.002339
2-oxoglutarate decarboxylation to succinyl-CoA	BioCyc	PWY-5084	2	9	0.002688
Glutathione metabolism	KEGG PATHWAY	ath00480	5	114	0.0039
alpha-Linolenic acid metabolism	KEGG PATHWAY	ath00592	3	39	0.005853
S-adenosyl-L-methionine biosynthesis	BioCyc	SAM-PWY	2	15	0.006465
ethylene biosynthesis I (plants)	BioCyc	ETHYL-PWY	2	20	0.010731
methionine degradation I (to homocysteine)	BioCyc	METHIONINE-DEG1-PWY	2	20	0.010731
Yang cycle	BioCyc	PWY-7270	2	25	0.015934
pyridine nucleotide cycling (plants)	BioCyc	PWY-5381	2	25	0.015934
betanidin degradation	BioCyc	PWY-5461	3	91	0.029949
baicalein degradation (hydrogen peroxide detoxification)	BioCyc	PWY-7214	3	91	0.029949
Phenylalanine biosynthesis	PANTHER	P02765	1	8	0.041622
glutamate biosynthesis II	BioCyc	GLUTAMATE-SYN2-PWY	1	5	0.042423
glutamate degradation I	BioCyc	GLUTAMATE-DEG1-PWY	1	5	0.042423
Plant-pathogen interaction	KEGG PATHWAY	ath04626	5	214	0.043299
Calvin-Benson-Bassham cycle	BioCyc	CALVIN-PWY	2	47	0.04828
Phenylalanine metabolism	KEGG PATHWAY	ath00360	4	155	0.050933
Carbon fixation in photosynthetic organisms	KEGG PATHWAY	ath00710	3	94	0.053298
glutamate biosynthesis III	BioCyc	GLUTSYNIII-PWY	1	7	0.056167
thiosulfate disproportionation III (rhodanese)	BioCyc	PWY-5350	1	8	0.062966
alanine degradation II (to D-lactate)	BioCyc	ALACAT2-PWY	1	8	0.062966
glutamate degradation IV	BioCyc	PWY-4321	1	8	0.062966
trehalose biosynthesis I	BioCyc	TRESYN-PWY	1	8	0.062966
Glutamine glutamate conversion	PANTHER	P02745	1	14	0.068592
coumarin biosynthesis (via 2-coumarate)	BioCyc	PWY-5176	2	58	0.069134
oxygenic photosynthesis	BioCyc	PHOTOALL-PWY	2	59	0.071155
gluconeogenesis I	BioCyc	GLUCONEO-	2	59	0.071155

		PWY			
superpathway of polyamine biosynthesis	BioCyc	PWYQT-94	1	12	0.089682
phenylalanine biosynthesis II	BioCyc	PWY-3462	1	12	0.089682
Plant hormone signal transduction	KEGG PATHWAY	ath04075	6	355	0.097157
anthocyanin biosynthesis (cyanidin 3-O-glucoside)	BioCyc	PWY-5125	1	14	0.102758
arginine biosynthesis IV	BioCyc	ARGININE-SYN4-PWY	1	14	0.102758
superpathway of phenylalanine and tyrosine biosynthesis	BioCyc	PWY-3481	1	14	0.102758
Degradation of aromatic compounds	KEGG PATHWAY	ath01220	1	12	0.108039
Cytoskeletal regulation by Rho GTPase	PANTHER	P00016	1	24	0.112189
Alanine, aspartate and glutamate metabolism	KEGG PATHWAY	ath00250	2	66	0.119378
methionine biosynthesis II	BioCyc	PWY-702	1	17	0.122026
Cysteine and methionine metabolism	KEGG PATHWAY	ath00270	3	138	0.126331
linoleate biosynthesis I (plants)	BioCyc	PWY-5995	1	19	0.134645
Taurine and hypotaurine metabolism	KEGG PATHWAY	ath00430	1	16	0.138892
Cyanoamino acid metabolism	KEGG PATHWAY	ath00460	2	73	0.139955
seleno-amino acid biosynthesis	BioCyc	PWY-6936	1	24	0.165416
photorespiration	BioCyc	PWY-181	1	24	0.165416
Starch and sucrose metabolism	KEGG PATHWAY	ath00500	4	244	0.172657
Selenocompound metabolism	KEGG PATHWAY	ath00450	1	21	0.175973
Butanoate metabolism	KEGG PATHWAY	ath00650	1	22	0.183197
Biosynthesis of amino acids	KEGG PATHWAY	ath01230	5	339	0.183623
Huntington disease	PANTHER	P00029	1	51	0.221804
superpathway of lysine, threonine and methionine biosynthesis II	BioCyc	PWY-724	1	35	0.229373
Photosynthesis - antenna proteins	KEGG PATHWAY	ath00196	1	30	0.238773
Arginine and proline metabolism	KEGG PATHWAY	ath00330	2	105	0.240686
Flavonoid biosynthesis	KEGG PATHWAY	ath00941	1	32	0.252071
Ubiquitin proteasome pathway	PANTHER	P00060	1	61	0.259536
superpathway of phenylalanine, tyrosine and tryptophan biosynthesis	BioCyc	COMPLETE-ARO-ARA-PWY	1	41	0.262201
Rubisco shunt	BioCyc	PWY-5723	1	45	0.283317
Biosynthesis of unsaturated fatty acids	KEGG PATHWAY	ath01040	1	43	0.321196
Ubiquinone and other terpenoid-quinone biosynthesis	KEGG PATHWAY	ath00130	1	43	0.321196
glycolysis I (from glucose-6P)	BioCyc	GLYCOLYSIS	1	54	0.328679

glycolysis II (from fructose-6P)	BioCyc	PWY-5484	1	55	0.333541
glycolysis IV (plant cytosol)	BioCyc	PWY-1042	1	56	0.338369
Biosynthesis of secondary metabolites	KEGG PATHWAY	ath01110	13	###	0.339905
beta-Alanine metabolism	KEGG PATHWAY	ath00410	1	50	0.361846
glycolysis VI (metazoan)	BioCyc	PWY66-400	1	61	0.361996
Metabolic pathways	KEGG PATHWAY	ath01100	23	###	0.373225
Nitrogen metabolism	KEGG PATHWAY	ath00910	1	54	0.383981
Fructose and mannose metabolism	KEGG PATHWAY	ath00051	1	63	0.43104
SNARE interactions in vesicular transport	KEGG PATHWAY	ath04130	1	69	0.460414
Stilbenoid, diarylheptanoid and gingerol biosynthesis	KEGG PATHWAY	ath00945	1	73	0.479156
Ubiquitin mediated proteolysis	KEGG PATHWAY	ath04120	2	183	0.48348
Carbon metabolism	KEGG PATHWAY	ath01200	3	309	0.516723
Phenylalanine, tyrosine and tryptophan biosynthesis	KEGG PATHWAY	ath00400	1	84	0.527434
superpathway of cytosolic glycolysis (plants), pyruvate dehydrogenase and TCA cycle	BioCyc	PWY-5464	1	105	0.537142
Fatty acid metabolism	KEGG PATHWAY	ath01212	1	88	0.543866
Inositol phosphate metabolism	KEGG PATHWAY	ath00562	1	88	0.543866
Glyoxylate and dicarboxylate metabolism	KEGG PATHWAY	ath00630	1	94	0.567459
superpathway of anaerobic sucrose degradation	BioCyc	PWY-7345	1	125	0.600188
Pyruvate metabolism	KEGG PATHWAY	ath00620	1	111	0.627932
RNA degradation	KEGG PATHWAY	ath03018	1	129	0.68284
Glycolysis / Gluconeogenesis	KEGG PATHWAY	ath00010	1	136	0.701952
Endocytosis	KEGG PATHWAY	ath04144	1	137	0.704588
Protein processing in endoplasmic reticulum	KEGG PATHWAY	ath04141	2	295	0.737294
RNA transport	KEGG PATHWAY	ath03013	1	220	0.858986
Ribosome	KEGG PATHWAY	ath03010	2	473	0.923802

Supplymentary table S3: Contigs related to flavour and colour from “Dashehari” transcriptome

Contig_ID	average DOI_FPKM	average D3I_FPKM	log2_foldchange	Gene Description
c13381_g1_i1	0.033268	5.448277	7.227558	abscisic acid 8'-hydroxylase 1-like [Citrus sinensis]
c27658_g4_i2	22.2182	497.4491	4.397324	putative 9-cis-epoxycarotenoid dioxygenase 3 [Citrus sinensis]
c27658_g4_i1	18.55559	329.0084	4.061596	putative 9-cis-epoxycarotenoid dioxygenase 3 [Citrus sinensis]
c27119_g1_i8	4.405029	63.70008	3.77005	phytoene synthase, partial [Mangifera indica]
c27119_g1_i6	3.51921	49.87668	3.741061	phytoene synthase, partial [Mangifera indica]
c27119_g1_i4	11.22784	141.3238	3.567338	phytoene synthase [Mangifera indica]
c27119_g1_i5	10.37392	129.5139	3.555545	phytoene synthase [Mangifera indica]
c27119_g1_i10	6.618453	82.53351	3.552361	phytoene synthase [Mangifera indica]
c27119_g1_i3	3.694458	45.60439	3.531677	phytoene synthase [Mangifera indica]
c27119_g1_i1	8.083365	98.42721	3.517494	phytoene synthase [Mangifera indica]
c27119_g1_i9	7.449599	86.88154	3.455692	phytoene synthase [Mangifera indica]
c11221_g1_i1	0	2.987052	2.838529	beta-carotene hydroxylase [Citrus sinensis]
c12919_g1_i1	0.143401	0.923558	2.628528	PREDICTED: carotenoid 9,10(9',10')-cleavage dioxygenase 1-like [Citrus sinensis]
c27119_g1_i7	0.501687	2.973282	2.473295	phytoene synthase, partial [Mangifera indica]
c27119_g1_i2	0.590074	3.273474	2.37304	phytoene synthase, partial [Mangifera indica]
c17148_g1_i1	0.321883	1.599115	2.206307	chalcone synthase [Rhus chinensis]
c17148_g1_i2	2.365269	10.23547	2.021087	chalcone synthases-1 [Mangifera indica]
c24941_g2_i1	26.74776	85.64991	1.591892	beta-carotene hydroxylase [Carica papaya]
c19583_g2_i1	20.09095	56.61051	1.407067	PREDICTED: leucoanthocyanidin reductase-like [Citrus sinensis]
c36578_g1_i1	0.318236	0	1.339231	lycopene epsilon cyclase [Citrus maxima]
c19583_g1_i1	1.063247	2.857554	1.331014	PREDICTED: leucoanthocyanidin reductase-like [Citrus sinensis]
c723_g1_i1	0.140308	0	1.312726	chalcone isomerase [Rhus chinensis]
c23384_g1_i2	6.011364	15.00728	1.232291	violaxanthin de-epoxidase [Citrus sinensis]
c24759_g6_i1	8.411842	20.03696	1.157457	PREDICTED: phytoene dehydrogenase, chloroplastic/chromoplastic isoform X2 [Citrus sinensis]
c23384_g1_i1	7.236825	17.14559	1.156587	violaxanthin de-epoxidase [Citrus sinensis]
c24759_g6_i2	5.922667	13.23694	1.06368	phytoene desaturase, partial [Manihot esculenta]

c8213_g1_i1	0	0.917503	1.04733	beta-carotene hydroxylase [Carica papaya]
c27000_g1_i6	0.895857	1.368048	0.518151	Chalcone-flavanone isomerase family protein isoform 3 [Theobroma cacao]
c24744_g1_i2	19.87391	29.33094	0.475203	zeta-carotene desaturase [Citrus unshiu]
c27429_g1_i6	12.99061	18.68446	0.437437	PREDICTED: carotenoid 9,10(9',10')-cleavage dioxygenase 1-like [Citrus sinensis]
c22798_g1_i1	8.97822	12.20959	0.3528	PREDICTED: prolycopene isomerase, chloroplastic isoform X1 [Citrus sinensis]
c24744_g1_i3	14.9935	18.6771	0.22779	zeta-carotene desaturase [Citrus unshiu]
c27429_g1_i1	2.095103	2.414979	0.096728	9-cis-epoxycarotenoid dioxygenase [Castanea mollissima]
c39008_g1_i1	0	0.37646	-0.0054	PREDICTED: chalcone synthase [Malus domestica]
c29453_g1_i1	0.866233	0	-0.08885	abscisic acid 8'-hydroxylase 3 [Arabidopsis thaliana]
c24320_g1_i1	0.188805	0.174696	-0.17404	PREDICTED: leucoanthocyanidin dioxygenase-like isoform X1 [Citrus sinensis]
c24320_g1_i2	0.18943	0.174842	-0.17613	PREDICTED: leucoanthocyanidin dioxygenase-like isoform X1 [Citrus sinensis]
c24320_g1_i3	0.190947	0.176241	-0.17613	PREDICTED: leucoanthocyanidin dioxygenase-like isoform X1 [Citrus sinensis]
c27429_g1_i2	47.8327	39.78068	-0.35133	PREDICTED: carotenoid 9,10(9',10')-cleavage dioxygenase 1-like [Citrus sinensis]
c27429_g1_i3	58.34967	47.22039	-0.38961	PREDICTED: carotenoid 9,10(9',10')-cleavage dioxygenase 1-like [Citrus sinensis]
c25999_g1_i1	3.812721	3.047875	-0.40491	Violaxanthin de-epoxidase-related [Theobroma cacao]
c16003_g2_i1	0	0.334472	-0.49515	PREDICTED: carotenoid 9,10(9',10')-cleavage dioxygenase 1-like isoform X2 [Citrus sinensis]
c27658_g2_i2	14.19335	10.21634	-0.55257	Nine-cis-epoxycarotenoid dioxygenase 3 [Theobroma cacao]
c14054_g1_i1	2.45272	1.732768	-0.59182	Nine-cis-epoxycarotenoid dioxygenase 4 [Theobroma cacao]
c25999_g1_i2	4.503446	3.160643	-0.59726	Violaxanthin de-epoxidase-related [Theobroma cacao]
c31038_g1_i1	0.907675	0.624953	-0.65986	anthocyanidin 5,3-O-glucosyltransferase-like
c19638_g2_i1	11.45054	7.373131	-0.72555	Leucoanthocyanidin reductase family protein
c20878_g1_i1	6.179518	3.67217	-0.83147	anthocyanidin 5,3-O-glucosyltransferase-like
c11756_g1_i1	7.07433	3.565891	-1.07576	phytoene synthase [Mangifera indica]
c24320_g1_i4	0.161165	0.073825	-1.18243	PREDICTED: leucoanthocyanidin dioxygenase-like isoform X1 [Citrus sinensis]
c48392_g1_i1	0.606417	0.277782	-1.18243	abscisic acid 8'-hydroxylase 3 [Arabidopsis thaliana]
c1082_g1_i1	2.379913	1.023636	-1.298	phytoene synthase, partial [Mangifera indica]
c16268_g1_i1	0	0.125108	-1.49515	PREDICTED: leucoanthocyanidin reductase-like [Citrus sinensis]

c15972_g1_i3	0.643163	0.23675	-1.52273	PREDICTED: abscisic acid 8'-hydroxylase 3-like [Glycine max]
c15972_g1_i4	0.669559	0.245173	-1.52914	PREDICTED: abscisic acid 8'-hydroxylase 3-like [Glycine max]
c15972_g1_i5	0.693057	0.253778	-1.52914	PREDICTED: abscisic acid 8'-hydroxylase 3-like [Glycine max]
c21978_g1_i1	5.270099	1.804064	-1.64947	PREDICTED: phytoene dehydrogenase, chloroplastic/chromoplastic-like [Citrus sinensis]
c26653_g1_i1	0.398278	0.121626	-1.76739	PREDICTED: zeaxanthin epoxidase, chloroplastic-like [Cicer arietinum]
c12919_g2_i1	0	0.127056	-2.38232	putative 9-cis-epoxycarotenoid dioxygenase, partial [Fagus sylvatica]
c38441_g1_i1	0	0.162501	-2.38232	chalcone synthase [Rhus chinensis]
c22556_g1_i1	8.029087	1.530216	-2.47563	PREDICTED: zeaxanthin epoxidase, chloroplastic-like [Citrus sinensis]
c53134_g1_i1	3.842426	0.586232	-2.85563	9-cis-epoxycarotenoid dioxygenase [Citrus hybrid cultivar]
c1330_g1_i1	12.50327	1.687759	-3.03228	9-cis-epoxycarotenoid dioxygenase [Citrus hybrid cultivar]

Supplementary Table S4: List of contigs in Dashehari transcriptome showing homology with ADH and HPPD.

**Contig_ID**	**Average DOI_FPKM**	**Average D3I_FPKM**	**log2_fold change**	**Gene Description**
c26529_g1_i2	7.636641834	11.64417558	0.521973	alcohol dehydrogenase 1 [Mangifera indica]
c26529_g1_i1	60.80832978	90.06892139	0.478147	alcohol dehydrogenase 2 [Mangifera indica]
c26529_g1_i3	90.1268097	131.429044	0.456376	alcohol dehydrogenase 2 [Mangifera indica]
c30227_g1_i1	1.191508926	0	-0.55197	alcohol dehydrogenase-like 6 [Arabidopsis thaliana]
c32724_g1_i1	0.794431151	0.270894655	-1.64776	putative alcohol dehydrogenase class III, partial [Solanum peruvianum]
c26565_g3_i1	30.96292952	15.08810691	-1.12597	PREDICTED: alcohol dehydrogenase class-3-like [Phoenix dactylifera]
c17466_g1_i1	1.840384101	2.760242087	0.485864	PREDICTED: alcohol dehydrogenase-like 6-like [Citrus sinensis]
c27898_g1_i3	252.6656047	120.6445444	-1.14848	p-hydroxyphenylpyruvate dioxygenase [Mangifera indica]
c27898_g1_i2	234.2128947	114.1504734	-1.12106	p-hydroxyphenylpyruvate dioxygenase [Mangifera indica]

Supplementary Table S5

Primers used for real time PCR validation

MiSAM s Fq (c27773_g1_i2)	TGCAACTGACGAGACACCTGA
MiSAM s Rq (c27773_g1_i2)	TGGGCAAGTTCCATTCTTGCG
MiACSFq (c18584)	CCCAGAAGCCTCTATCTGCAC
MiACSRq (c18584)	TTCTGAACTCTGGCAAGCCG
MiACOFq (c22984_g1_i3)	TCC CTA TAA ATA CCA CCC CAA CCT CC
MiACORq (c22984_g1_i3)	CTC AAG CTC TCC ATC TCT CAC AAA TGG
MiEIN3_2495Fq	TCTACCGAAACCCGATATTCT
MiEIN3_2495Rq	GATGACAAAACGAGAGAGAACT
MiEIN3_2525Fq	CTACGGGTTGCTTTTTCGG
MiEIN3_2525Rq	TCTTTTCTCTTCGACTTGGTTG
MiETRRq (c29681_g1_i1)	ATCAGCTGGCCATTGTGGT
MiETRFq (c29681_g1_i1)	TCTCATGAATTGTTCACGGGCTTA
MiExpFq (c25255)	TCCCAGTTTCATACCGCCGC
MiExpRq (c25255)	ATGTCTCCTGAACCCGCCAC
MiPGFq (c26690)	GAAAGCTGGTGGAAGAATG
MiPGRq (c26690)	CTGTTGACGTGATGCAATCC
MiCelFq (c27942)	CAATGCTCCTCTCCTGGGTA
MiCelRq (c27942)	AATTGGATTCGACGAAGCTG
MiPelFq (c27147)	GCTTAGGGGCAAAATCCTCT
MiPelRq (c27147)	ACGGGAGTTGTGGTGTTGAT
MiActFq	GTGGTCGTACAACTGGTATTGTGCT
MiActRq	TTGCATGTGGGAGTGCATAACCCT
